# Recent advances in CRISPR technologies for biomedical applications and disease treatment

**DOI:** 10.7150/thno.136062

**Published:** 2026-09-24

**Authors:** Xue Wu, Xiaorong Kou, Liping Bai, Chao Liu, Qinjie Wu, Ming Zhang, Ning Wang, Changyang Gong

**Affiliations:** 1 Department of Ophthalmology, West China Hospital, Sichuan University, Chengdu, 610041, China.; 2 Department of Biotherapy, Cancer Center and State Key Laboratory of Biotherapy, West China Hospital, Sichuan University, Chengdu, 610041, China.; 3 Department of Otolaryngology-Head &Neck Surgery, West China Hospital, Sichuan University, Chengdu, 610041, China.

**Keywords:** CRISPR, genome editing, biomedical research, disease treatment, delivery system

## Abstract

Clustered regularly interspaced short palindromic repeats (CRISPR), a precise genome-editing platform, has revolutionized genetic engineering. The highly selective and programmable characteristics of CRISPR have enabled its broad application. Recent advances in CRISPR biotechnology have promoted its widespread use in various biomedical fields, such as CRISPR screening. Ongoing preclinical and clinical trials will help determine the full capabilities of CRISPR in disease therapy. The efficacy of CRISPR-based applications is fundamentally dependent on optimized delivery strategies. Multiple approaches for delivering the CRISPR components have been investigated, primarily relying on physical, viral and non-viral delivery methods. Each method has specific strengths and limitations. Notably, non-viral delivery systems hold significant promise for CRISPR applications, offering distinct advantages such as transient expression, low immunogenicity, and fewer size constraints for cargo delivery. Various biomaterials demonstrate strong potential for delivering CRISPR components to achieve gene editing, including inorganic nanoparticles, polymeric nanoparticles, lipid nanoparticles, metal–organic frameworks, DNA origami, and virus-like particles. Therefore, this review provides an overview of recent research on the molecular principles of CRISPR and its biomedical applications. Recent advances in CRISPR-based treatments and their delivery vectors are also reviewed. Finally, the challenges faced by CRISPR technology are discussed.

## 1. Introduction

Genome editing has been widely exploited not only to prevent or alleviate disorders resulting from gene mutations but also to explore pathogenic biomolecular processes. The major available genome-editing platforms encompass zinc finger nuclease (ZFN), transcription activator-like effector nuclease (TALEN), and clustered regularly interspaced short palindromic repeats (CRISPR) systems 1. The CRISPR technology is a novel nuclease system originally discovered in bacteria that protects microorganisms from bacteriophages by cleaving foreign nucleic acid sequences 2. Furthermore, CRISPR system has been optimized for widespread application in fundamental biological research (Figure 1) 3-16. CRISPR components include a CRISPR-associated protein system (Cas) effector for nuclease activity and single-guide RNA (sgRNA) for specific targeting 17. Cas effectors can be more easily customized for specific targets than conventional gene editing systems such as TALEN and ZFN 18. Diverse CRISPR effector proteins with various sizes, structures, compositions and targeting mechanisms have been discovered. These discoveries have greatly broadened the utilization of CRISPR for genome editing and laid the foundation for its clinical application.

Recently, the highly selective and programmable mechanisms of CRISPR have facilitated widespread utilization across diverse biological and medical applications. Beyond classical application, base editing systems can effectively correct single-base mutations 17. Unlike nuclease-based methods, base editing does not introduce double-strand breaks (DSBs). In addition, the newly developed CRISPR prime editing can theoretically produce all possible base substitutions 13. Prime editing also introduces targeted mutations without DSBs and may result in insertions of up to 44 bp via complementary prime editing guide RNA (pegRNA) 13. Furthermore, CRISPR-based genetic tools, such as CRISPR activation (CRISPRa) and CRISPR interference (CRISPRi), also exhibit remarkable specificity 19. Overall, the flexibility and simplicity of CRISPR system have enabled its wide employment in various biomedical fields, including CRISPR screening and drug resistance research, CRISPR/Cas nucleic acid detection, and disease model construction 20-23. These advancements have accelerated basic research and spurred innovation across the life sciences.

Progress in CRISPR technology has driven innovations in biological research and illustrated enormous therapeutic capacity 14,24. Notably, CRISPR has demonstrated remarkable utilization across various diseases, including hematological, eye-related, metabolic, neurodegenerative, and autoimmune diseases, as well as other intractable disorders. Ongoing preclinical and clinical trials will help determine the full capabilities of CRISPR 24. The first-in-human phase I clinical trial of editing PD-1 in T cells to treat lung cancer demonstrated the clinical application of CRISPR technology 11. Moreover, Casgevy (exagamglogene autotemcel) has been authorized for managing patients with either sickle cell disease (SCD) or transfusion dependent β-thalassemia (TDT) 14,25-27. Furthermore, CRISPR delivery strategies play an important role in clinical application. The main methods for delivering CRISPR components include physical, viral and non-viral delivery approaches 2,20,28-33. Each approach presents a unique profile of strengths and limitations. Physical strategies can deliver CRISPR components by penetrating cellular barriers 20,28. Viral vectors, particularly adeno-associated viruses (AAVs), exhibit superior delivery efficiency and tissue-specific tropism, but encounter obstacles including immunogenicity and restricted cargo capacity. In contrast, non-viral delivery systems provide enhanced biosafety by circumventing immune responses and can be modified to improve delivery precision 29-33. Optimizing these delivery systems is pivotal for advancing CRISPR toward clinical translation.

While its advantages are undeniable, several challenges remain in CRISPR-based treatments, such as off-target effects and ethical issues 34,35. Current research focuses on refining CRISPR specificity and developing novel strategies to overcome its limitations. Therefore, this review presents an overview of recent research on the molecular principles and biomedical applications of CRISPR. Recent advances in CRISPR-based treatments and their delivery vectors are also reviewed. Finally, the challenges encountered by CRISPR technology are discussed.

## 2. Different types and mechanisms of CRISPR technology

### 2.1. CRISPR/Cas effector proteins

CRISPR/Cas effector proteins can be classified into six types (I-VI), each with a characteristic Cas nuclease component, which are grouped into two kinds (class I or class II). Specifically, Class I systems (types I, III, and IV) rely on multi-subunit Cas effector proteins and can be employed for eukaryotic gene editing, but their complexity has restricted their widespread application 36-38. Nevertheless, Class II systems, including type II (Cas9), type V (Cas12), and type VI (Cas13), utilize a single Cas effector protein to accomplish targeted RNA or DNA cleavage, thus exhibiting enormous promise for gene editing 38. Among them, Cas9 derived from *Streptococcus pyogenes* has been broadly utilized and can generate DSBs at precise genomic loci using programmable gRNA molecules 39. Meanwhile, Cas12a utilizes a single crRNA with a defined protospacer-adjacent motif (PAM), exhibiting high specificity, low off-target effects and strong potential for nucleic acid detection 40-46. In contrast, Cas13a/b targets RNA without a strict PAM requirement, primarily facilitating effective post-transcriptional knockdown 46-50. The main features of these class II systems have been summarized in Table 1.

Notably, the miniaturization of enzymes in CRISPR technology has emerged as a pivotal optimization strategy. The Cas12 family has several compact nucleases, such as CasX (Cas12e, <1000 amino acids) and Cas12f (Cas14, 400–700 amino acids) 52,53. The DNA cleavage properties, compact size, and non-pathogenic origin of CasX enzyme confer significant advantages over other CRISPR/Cas genome-editing enzymes 52. Cas12f nuclease is among the smallest genome-editing tools currently available and has advantages in efficient delivery via size-limited delivery vectors such as AAV 53. Similar to other Cas12 nucleases, Cas12f can cleave nonspecific ssDNA upon binding to complementary target DNA, making it a promising tool for nucleic acid detection 42,54. In addition, compared with Cas9 and Cas12a nucleases, Cas12f nuclease markedly reduces large deletions and chromosomal translocations 55. However, the gene-editing activity of Cas12f is inferior to that of the widely used Cas9 and Cas12a 56. Notably, following Cas effector-mediated cleavage, the resulting DSB is repaired by the endogenous DNA repair mechanisms, which ultimately dictate the final outcome of genome editing.

### 2.2. DNA repair mechanisms for genome editing

The repair of DSBs is mediated predominantly by two distinct pathways, specifically non-homologous end joining (NHEJ) and homology-directed repair (HDR). As a versatile genome engineering tool, CRISPR technology leverages these mechanisms to achieve targeted genetic modifications. Specifically, three major genome-editing platforms, namely ZFN, TALEN, and CRISPR/Cas system, all of which function by generating targeted DSBs at predefined genomic loci, are depicted in Figure 2A. Notably, unlike ZFN and TALEN that rely on direct protein‒DNA interactions for target recognition, CRISPR/Cas system achieves sequence specificity primarily via programmable base pairing between sgRNA and target DNA, thereby introducing targeted DSBs 9,57. With respect to the downstream repair processes, the two major DSB repair pathways are depicted in Figure 2B, where NHEJ pathway ligates the two ends of a DSB and predominates as an efficient repair mechanism, whereas HDR pathway utilizes a homologous donor template to achieve precise repair. Consequently, the NHEJ pathway introduces insertions or deletions (indels) without requiring a homologous donor, which disrupts protein-coding sequences to achieve functional gene knockout 2,17,58,59, which remains the most widely utilized CRISPR application and serves as the foundation for loss-of-function studies. In contrast, as an alternative pathway to NHEJ, the HDR pathway allows cells to repair DSBs by introducing exogenous template DNA 17,60. Specifically, this procedure is significantly more effective for inserting foreign gene sequences at a site when the target DSB is introduced using Cas effectors. With this approach, disease-causing mutations can be repaired. Nevertheless, although HDR can precisely facilitate targeted gene modification, it is generally less efficient than NHEJ which does not require a DNA template to generate insertions or deletions 20.

### 2.3. Base editing

Owing to the inefficiency of HDR and the indeterminacy of NHEJ, base editing systems possess a considerable ability to correct single-base mutations 17. Unlike nuclease-based methods, base editing does not introduce DSBs. Thus, base editing system provides a targeted and significantly effective method for correcting SNPs related to specific disorders 17. The base editing system primarily comprises two types: adenine base editor (ABE) and cytosine base editor (CBE). The ABE system comprises sgRNA and adenosine deaminase-fused nickase Cas9 (nCas9), resulting in effective base transitions with a lower occurrence of indels 17. In contrast, CBE system utilizes a cytidine deaminase-fused nCas9 to cause C·G-to-T·A editing at target gene loci 12. With the development of basic research, base editing systems have become more flexible and effective 61. However, the mutagenic properties of base editors have raised concerns about off-target effects. Therefore, improvements in base editing system need to focus on providing accurate single nucleotide editing. While base editing achieves precise single nucleotide transition with high efficiency, it is constrained by the limited base transitions, which cannot meet the repair needs of all single nucleotide variants. These limitations motivated the development of prime editing, which utilizes an engineered nCas9 fused to reverse transcriptase, enabling small insertions, deletions and base conversions without inducing DSBs or requiring exogenous donor DNA templates 62.

### 2.4. Prime editing

Notably, recently developed CRISPR tools, known as prime editing tools, can theoretically produce all possible base substitutions 13. Table 2 summarizes the key characteristics of base editing and prime editing systems. Mechanistically, prime editing variants generally exploit nCas9 fused with reverse transcriptase and utilize pegRNA 24. Specifically, pegRNAs are composed of sgRNA scaffold with a 3' extension that provides a reverse transcription template, thereby creating specific alterations at the target site 63. Prime editing introduces targeted mutations in the absence of DSBs and may result in insertions of up to 44 bp via complementary pegRNAs 13. Third-generation prime editing systems, represented by PE3 and PE3b, utilize a supplementary sgRNA to nick the non-edited strand 24, which greatly improves editing efficacy 13. Building on these advances, a prime editing therapy for chronic granulomatous disease was cleared for clinical trials in 2024, representing a pivotal milestone in the clinical translation of prime editing 15.

### 2.5. Chemical and physical control

Recently, chemical and physical methods for modulating the CRISPR system have achieved promising outcomes in enhancing spatiotemporal controllability. Commonly used chemical and physical strategies include small-molecule induction, thermal modulation, ultrasonic activation and optical regulation of the CRISPR system 65-68. Doxycycline and rapamycin are representative small molecules for chemical regulation 69-71; however, they tend to diffuse freely and may have toxic side effects. Moreover, thermal modulation affects intracellular biological processes and is difficult to control precisely, while ultrasound displays limited penetration capacity in cells and tissues 72. In contrast to these alternative approaches, optical regulation of Cas9 function exhibits distinct advantages in terms of non-invasiveness, spatiotemporal specificity, and reversibility 73. Nevertheless, a prevalent drawback of such gene regulatory systems is potential leakage, characterized by residual activity under non-illuminated conditions. This issue can be alleviated by optimizing the interaction interfaces between effector proteins and photosensitive elements 74. Additionally, inefficient penetration of deep tissue during *in vivo* applications remains a key challenge for current photoactivation-based strategies 75. Collectively, each chemical and physical modulation strategy involves unique benefits and drawbacks. Therefore, the selection of an optimal modulation strategy should be tailored to the specific demands of the experimental setting or therapeutic application.

## 3. CRISPR epigenome editing

Based on evolved CRISPR platforms, significant progress has been made in the development of epigenome editing tools, including CRISPRa and CRISPRi, DNA methylation and histone modification editing, as well as integrated systems such as CRISPRon and CRISPRoff. Notably, unlike the genome editing tools shown in Figure 3A-C, epigenome editing tools typically utilize catalytically dead Cas9 (dCas9) fused to diverse effectors to precisely control gene transcription and epigenetic marks, leaving the DNA sequence unchanged 76.

### 3.1. CRISPR activation and interference

Apart from the typical CRISPR application of targeted gene knockout, CRISPRa and CRISPRi represent the most widely used epigenome editing tools, which enable targeted transcriptional activation and repression, respectively (Figure 3D-E) 77. Specifically, Table 3 presents representative examples of gene activation and inhibition via CRISPRa and CRISPRi technology. Moreover, transcriptional regulation by CRISPRa and CRISPRi is utilized in a broad spectrum of biomedical applications, such as genetic screening, gene function studies, and preclinical research on hereditary diseases 77,78.

#### 3.1.1. CRISPRa

Gene activation offers crucial phenotypic knowledge and may be influenced by multiple aspects, including cell type, basic level of gene expression, sgRNA efficiency, and epigenetic characteristics 79,80. Notably, a threefold increase in basal gene expression levels can be effective for certain phenotypes, while some phenotypes require higher expression levels. Whether a universal dCas9 effector can achieve general and effective activation in different cell types and gene targets remains to be explored 81. The single activation domain commonly used during early research was VP64 10,82. On the basis of existing technologies, other alternative single-activation domain systems, including those involving VP160, p300, and CREB-binding protein (CBP), continue to be developed 83,84. VP160 and VP64 similarly promote the upregulation of target genes 85. However, the p300 domain substantially increased gene activation by up to two times that of VP64 in HEK293T cells 83. Similarly, the CBP domain can induce more than twofold activation of some targets relative to the combinatorial synergistic activation mediator system 86.

Furthermore, CRISPRa is a promising approach that can reveal the complex role of target genes in cell differentiation and has a major influence on effective disease therapy. For example, CRISPRa targeting the Ngn2 and Isl1 genes resulted in specialized differentiation of mouse embryonic fibroblasts or astrocytes into motor neurons 87*.* With respect to the cardiac abnormalities caused by dystrophin deficiency, mortality in mice with heart failure was effectively reduced by tail-vein injection of dCas9-VP64 packaged with AAV9 to activate dystrophin 88. However, the CRISPRa system may be unsuitable for a single viral delivery approach because of its larger size relative to that of Cas9 89. As alternatives to AAVs, nanoparticles are capable of delivering the dCas9 system to targeted sites, activating tumor suppressor factors *in situ* to effectively inhibit tumor progression 90.

#### 3.1.2. CRISPRi

The CRISPRi system originally utilized the KRAB domain as a transcriptional repressor to achieve powerful transcriptional inhibition 91. Owing to its greater specificity, CRISPRi can exhibit more efficient suppression when it targets miRNA clusters than short hairpin RNA (shRNA) and short interfering RNA (siRNA) 92. The inhibition of miRNAs is promising for treating specific malignant tumors with high miRNA expression levels 77. The development of novel drug discovery methods can be enhanced and accelerated by the CRISPRi system, facilitating the evolution of innovative treatments. For example, CRISPRi is currently being used in preclinical studies of retinal diseases. Retinitis pigmentosa, which is a hereditary disease that results in severe photoreceptor degeneration, can be treated in animal models by using KRAB-dCas9 93. In this therapy, CRISPRi components were delivered by subretinal injection of AAV vectors and inhibited Nrl gene expression, which reprogrammed the rods into cone-like cells. These results demonstrated that inhibiting Nrl gene expression rescued the degradation of photoreceptors and demonstrated significant potential for the treatment of retinitis pigmentosa. However, much remains to be further explored in terms of the fundamental characteristics and innovative applications of the CRISPRi system.

### 3.2. DNA methylation and histone modification editing

DNA methylation and histone modification constitute the two predominant mechanisms of epigenetic regulation. DNA methylation at promoter CpG dinucleotides generally leads to transcriptional silencing by hindering transcription factor binding and promoting a compact chromatin structure, which is predominantly mediated by the DNA methyltransferase (DNMT) family 98. Specifically, DNMT1 maintains established methylation patterns and DNMT3A/3B catalyze *de novo* methylation, whereas ten-eleven translocation (TET) enzymes mediate active demethylation via 5-methylcytosine oxidation 99-102. The effect of histone modifications on gene expression is either activating or repressive, with the functional outcome dictated by the modification type 103. For instance, histone methyltransferases and demethylases dynamically regulate methylation, while acetyltransferases and histone deacetylases (HDACs) similarly control acetylation. Notably, the removal of acetyl groups by HDACs typically induces chromatin compaction, thereby leading to transcriptional repression 104. Leveraging these regulatory mechanisms, recent studies have successfully achieved histone modifications by fusing dCas9 to various effector enzymes, notably LSD1, G9A, EZH2, and P300 14,83. Collectively, these advances highlight the therapeutic potential of epigenetic modifiers to correct aberrant gene expression patterns in intractable diseases, such as cancer, neurodegenerative disorders, and metabolic syndromes 76.

### 3.3. CRISPRon and CRISPRoff

Epigenome editing systems integrating multiple epigenetic effectors exhibit superior efficacy in achieving stable and robust gene regulation. Notably, the development of CRISPRon and CRISPRoff platforms, which integrate multiple epigenetic effector domains, represents a landmark advance 105,106. CRISPRoff system establishes heritable long-term gene silencing via targeted DNA methylation that persists across cell divisions. Correspondingly, CRISPRon can reverse this silencing through targeted DNA demethylation, thereby offering unprecedented reversibility and tunable control over epigenetic landscapes 105. Unlike single-component effectors, such as dCas9-DNMT3A-DNMT3L and dCas9-KRAB, CRISPRoff combines the DNMT3A and DNMT3L effector domains with the KRAB repression domain, thereby achieving epigenetic silencing with significantly enhanced durability and stability 105,106. In contrast, CRISPRon system achieves reversal of CRISPRoff-mediated epigenetic silencing by fusing dCas9 to the TET1 catalytic domain paired with engineered sgRNAs, thereby restoring transcriptional activity 105. Using a dCas9-TET demethylation tool, researchers reversed the durable PCSK9 silencing imposed by epigenetic editors, effectively reactivating transcription 107. This inherent tunability could theoretically mitigate the long-term risks associated with irreversible genome editing, representing a promising strategy for managing chronic diseases that demand adjustable therapeutic interventions.

## 4. CRISPR screening and drug resistance research

CRISPR-based high-throughput screening has emerged as a cornerstone of functional genomics due to its programmability and flexibility. Unlike RNA interference (RNAi) screening, which relies on partial post-transcriptional silencing and is characterized by variable knockdown efficiency and frequent off-target binding to partially complementary transcripts, CRISPR-based screening generates defined genetic alterations at the DNA level with superior consistency and lower off-target effects 108,109. Specifically, CRISPR off-target activity is inherently constrained by PAM recognition and can be further mitigated by using high-fidelity Cas variants and optimized sgRNA design. CRISPR screening has been deployed across a continuum of experimental models. *In vitro* screening offers unparalleled capacity and experimental controllability but fails to recapitulate the complex tissue architecture. Indirect* in vivo* screening partially addresses this limitation by introducing sgRNA libraries into cultured cells prior to murine transplantation, thereby recapitulating *in vivo* physiological and pathological processes and enabling the investigation of oncogenes 110, tumor suppressors 111-113, synthetic lethal genes 114, and immunotherapy regulators 115. However, transplanted cells remain constrained by organ microenvironment 116. Direct *in vivo* screening circumvents these constraints by inducing mutagenesis directly in target tissues while preserving the microenvironment and immune function 116, as exemplified by intracranial injection of AAV carrying sgRNA achieving direct mutagenesis in the animal brain 117. Nevertheless, the capacity of direct *in vivo* CRISPR screening to faithfully recapitulate the pathogenesis of diverse disorders warrants further systematic evaluation.

By utilizing customized sgRNA libraries, CRISPR screening can simultaneously assess multiple genomic components, including protein-coding genes, noncoding RNAs and enhancers 118, facilitating unbiased identification of regulators driving specific phenotypes such as tumorigenesis 116. Notably, this approach has revealed therapeutic targets, including CMTM6 as a candidate antitumor target 119 and POR as a regulator of ferroptosis 120. Furthermore, a customized miRKOv2 library was developed to identify miRNAs essential for prostate cancer cell survival through CRISPR screening (Figure 4A) 121. Beyond target discovery, CRISPR-based findings can guide drug development by directing therapeutic agents against pathogenic genes to disrupt critical biological pathways and selectively inhibit malignant cells 21,122,123. Key antioncogenes (TP53 and PTEN) and oncogenes (MYC, KRAS, and EGFR) have been identified via CRISPR technology as promising drug targets 124. However, translating CRISPR-based discoveries into clinically efficacious therapeutics remains challenging, owing to off-target effects, inefficient gene editing, and the necessity for rigorous preclinical and clinical validation 124,125.

Drug resistance research represents another major application of CRISPR screening. Gene mutations are important drivers of acquired drug resistance 126, and CRISPR screening enables systematic identification of resistance-associated genes. For example, a genome-wide CRISPR screening in rSKBR3 cells employing a lentiviral library of 123411 sgRNAs successfully identified anti-HER2 resistance genes through integrated *in vitro* and *in vivo* approaches (Figure 4B) 127. Similarly, CRISPR screening in chronic myeloid leukemia cells uncovered gene perturbation profiles that mediate drug resistance and sensitization to tyrosine kinase inhibitors and other therapeutic agents, as reflected by relative sgRNA enrichment or depletion 128. Furthermore, CRISPR/Cas9 screening demonstrated that USP1 conferred resistance to cisplatin treatment (Figure 4C) 129. Collectively, these studies illustrate the utility of CRISPR screening in identifying resistance mutations to facilitate targeted drug development. Overall, candidate genes and drug targets for disease treatment can be identified by CRISPR screening. However, downstream regulatory mechanisms require further characterization, and the development of efficacious medications directed at these targets remains challenging, as the comprehensive functions of the identified genes cannot be fully elucidated owing to the inherent stochasticity of experimental procedures 130.

## 5. CRISPR/Cas nucleic acid detection

Pathogen nucleic acid detection based on genetic elements enables timely diagnosis and therapy. Nevertheless, such diagnostics rely on technologies that can detect trace concentrations of nucleic acids with excellent specificity and sensitivity 131. In this review, current advancements in CRISPR/Cas nucleic acid detection are discussed.

Approaches that facilitate the specific, sensitive, and rapid analysis of nucleic acid sequences contribute greatly to accurate disease diagnosis and efficient clinical therapy 132. Established genomic diagnostic approaches, including polymerase chain reaction (PCR), demonstrate advantages in amplifying genomic sequences. However, they have drawbacks in terms of practical utility and cost 133. Most existing nucleic acid detection methods require complicated and cumbersome instruments, in addition to skilled staff. Owing to multiple manual steps that take time and materials, the cost of detection is high 39. Isothermal amplification methods are quicker than PCR. They avoid the need for complex devices such as thermocyclers and can be performed at a stable temperature 134. Nevertheless, these emerging benefits entail the sacrifice of specificity and sensitivity. Despite efforts toward optimization, SNPs are not always able to be accurately distinguished 135, although the ability to detect changes in small nucleotides is critical in the identification of pathogens and diseases. Currently, the demand for time-efficient and cost-effective nucleic acid testing methods is still growing in fields such as genotyping and pathogen detection. Consequently, nucleic acid-based diagnostics must combine the flexibility, specificity, and sensitivity of existing genomic diagnostic methods with the usability, speed, and cost efficiency of isothermal amplification approaches 136. Compared with existing nucleic acid detection methods, CRISPR/Cas-based systems offer promising alternatives (Table 4).

In general, CRISPR-based biosensing with isothermal signal amplification and high base resolution promises to be a remarkable approach to meet the demands of ultrasensitive nucleotide detection, including SNP identification, cancer mutation testing, human genotyping and pathogen detection, especially in point-of-care diagnostic applications 137-139. In recent decades, the unique properties of Cas proteins, such as Cas12 and Cas13, have enabled excellent sensitivity and selectivity for nucleotide detection 140. In addition to DNA and RNA, CRISPR-based sensing can also contribute to the detection of small molecules and proteins 138,140. Research has indicated that CRISPR biosensing methods may be adjusted and modified within a few days to identify specific viruses, thus enabling diagnostic preparation for future pandemics 39. Owing to their increased accuracy and sensitivity, optimal quick diagnostic methods can be used in multiple clinical applications. As a result, CRISPR technology is highly important for the rapid and specific diagnosis and treatment of infectious diseases 141.

## 6. Disease model construction

### 6.1. Human stem cells and organoids

Integrating stem cell treatment with CRISPR-based genome editing is highly promising for managing multiple genetic disorders. Disorders resulting from monogenic mutation, including SCD, muscular dystrophy and cystic fibrosis, may benefit from this strategy 142. For example, transplanting the functional stem cells produced via CRISPR technology into patients has demonstrated the potential to restore normal blood cells and reduce the clinical manifestations of SCD 143. Human pluripotent stem cells (hPSCs) can also be treated using CRISPR technology for applications in multiple fields such as cell therapy and drug screening, thereby addressing the limitations of hPSCs (Figure 5) 144-146. The application of precise gene modification of hPSCs can contribute to regenerative medicine 144,146 and the analysis of the genetic differences between different types of cells and tissues. In addition, the CRISPR/Cas9 technique seems to be significantly effective and precise for hPSC gene editing and has various benefits over previous approaches, including ZFN and TALEN 147,148. Nevertheless, each disease is associated with unique challenges that require careful assessment to determine the appropriateness of genome editing via CRISPR and subsequent stem cell treatment 149. Moreover, ethical concerns must also be considered carefully. Therefore, rigorous ethical principles to confirm that genome editing is performed conscientiously are necessary 142.

Organoid technology allows the three-dimensional reconstruction of multiple tissues from stem cells that recapitulate important tissue phenotypes 150 and can be used as disease models. CRISPR technology works well in genetically engineering organoids for the study of single-gene diseases and cancers 151. The use of organoid culture for tumor modeling represents the latest advance in drug discovery. As an alternative strategy for optimizing treatment regimens, researchers have leveraged the ability of organoids to expand tissues directly and use them for drug screening (Figure 5) 152-154. In one study, cancer organoids with different genotypes presented different drug sensitivities and reproduced predicted responses 155. Drug screening based on organoids may contribute to clinical decision-making in terms of choosing effective therapies and avoiding unnecessary administration of ineffective drugs.

### 6.2. Engineered animal model

The most prominent application of CRISPR technology involves the development of engineered animal models to investigate pathogenic mechanisms and evaluate treatment strategies. Previously preferred method for genome modification, gene targeting of homologous recombinant embryonic stem cells, seems to be high-cost and requires the establishment of cell lines 156. CRISPR system overcomes these obstacles and is currently routinely applied to produce gene mutations in mice 157,158. The modification of zygotes by CRISPR is particularly powerful for editing multiple targets simultaneously or for introducing novel alterations into current animal models with various transgenic alleles 158. Provided that appropriate gRNAs are selected, CRISPR/Cas9 gene targeting may be accomplished in different murine backgrounds. In addition, Cas9 ribonucleoprotein complex (RNP) electroporation of mouse zygotes has been achieved, eliminating the need for laborious procedures 159,160. Although this gene editing method is extremely effective, more investigations are needed to optimize the HDR pathway for larger templates. In summary, innovative strategies promote the development of engineered animal models. In addition to its use in germline editing, CRISPR technology may also be utilized for modifying somatic cells. To promote the use of CRISPR technology in somatic cells and mouse models, transgenic mice with constitutive, conditional, or inducible Cas9 expression have been produced 161-163. Besides, sgRNA sequences can be transduced in pools or individually to modify cells in transgenic mice expressing Cas9. In a recent study, Cas9 RNP electroporation was effectively utilized to directly modify primary immune cells in mice 164. This strategy facilitates the assessment of phenotypes caused by specific perturbations in immune cells 164.

## 7. CRISPR technology in various disease treatments

CRISPR technology has contributed greatly to disease treatment (Table 5), including hematological, eye-related, metabolic, neurodegenerative, and autoimmune diseases, as well as other intractable disorders 165-168. Ongoing preclinical and clinical trials will help determine the full capabilities of CRISPR 24. The pace of progress in CRISPR-based disease therapy is determined by multiple factors. Specifically, compared with polygenic conditions (e.g., autoimmune diseases), in which pathogenic mechanisms remain incompletely elucidated and therapeutic outcomes are less predictable, monogenic disorders with well-characterized causal mutations (e.g., SCD and TDT) are more amenable to CRISPR-mediated gene correction 26,169. Another critical determinant is targeting cell accessibility. Diseases affecting easily accessible or *ex vivo* manipulable cells (e.g., hematopoietic stem cells in β-hemoglo-binopathies) have progressed more rapidly than those requiring delivery across the blood–brain barrier (e.g., Huntington’s disease) 26,170. Moreover, the availability of effective delivery systems plays a critical role. Organs with clinically validated delivery platforms (e.g., the liver via lipid nanoparticle, the retina and choroid via subretinal AAV) have facilitated significantly faster clinical translation compared to organs lacking efficient and safe delivery strategies (e.g., the lung and pancreas) 171,172. Building on this framework, the following discussion systematically elaborates on the research progress of CRISPR-based therapies for each disease category.

### 7.1. Hematologic diseases

#### β-Hemoglobinopathy

β-Hemoglobinopathy, which affects the β chain of hemoglobin, represents the most prevalent monogenic disorder and manifests mainly in the form of SCD and TDT 173. SCD is a single-gene disease resulting from β-globin gene mutation that causes ischemic tissue injury‌ and organ failure 174. β-Thalassemia, by contrast, can be attributable to multiple mutations resulting in decreased production of functional β-globin, which in turn causes impaired erythropoiesis and hemolysis. Regardless of‌ the differences in underlying pathology, mutations that increase the level of fetal hemoglobin (HbF) can ameliorate both SCD and TDT because γ-globin has antisickle ‌characteristics and is capable of replacing the function of β-globin 173. Therefore, several strategies involving CRISPR technology for treating β-hemoglobinopathies are dependent on the reactivation of γ-globin 63.

Recently, many clinical trials have aimed to address β-hemoglobinopathies using different CRISPR-based tools. For example, CTX001 is a CRISPR-mediated autologous progenitor cell therapy for TDT and SCD (ClinicalTrials.gov Identifiers: NCT03655678 and NCT03745287) 26. Exagam-glogene autotemcel (Casgevy) for treating SCD and TDT is a major milestone for CRISPR in the clinic 14,25-27. In general, CRISPR-based gene therapy can function as a promising strategy for treating β-hemoglobinopathy. In addition, the great progress involving CRISPR has laid the foundation for this breakthrough technology to be applied more widely to other genetic diseases.

#### Hemophilia

Hemophilia A is attributable to FVIII gene mutations, and hemophilia B derives from FIX gene mutations 175. Gene therapy is regarded as the most promising approach for hemophilia therapy, inducing endogenous synthesis of FVIII or FIX 175. A promising gene therapy approach utilizes CRISPR/Cas technology 176. In an *ex vivo* study, the germline cells of hemophilia B mice were injected with three different Cas9 variants to evaluate the effectiveness and safety of genetic correction 177. In addition, Tang *et al*. integrated the active FIX variant into human iPSCs *in vitro* using CRISPR technology 178. The results revealed that the FIX activity was increased by 4.2-fold 178. Gene therapy by CRISPR technology provides valuable perspectives for research on hemophilia treatment.

### 7.2. Eye-related diseases

Leber congenital amaurosis 10 represents a hereditary retinal disorder induced by CEP290 gene mutations that cause severe dystrophy of photoreceptor cells and poor vision in early infancy 179. A single-dose escalation clinical trial of EDIT-101 to treat Leber congenital amaurosis 10 was performed in 2019 (ClinicalTrials.gov identifier: NCT03872479) 180. EDIT-101 represents a potential genome editing treatment in which Cas9 and sgRNA are delivered by the AAV5 vector via subretinal injection 180. The treatment can target photoreceptor cells to correct CEP290 gene mutations and restore the functional expression of CEP290 181. Although anti-VEGF drugs are beneficial for decreasing ocular neovascularization, multiple challenges still need to be considered 182. Genome editing of VEGFR2* in vivo* through CRISPR/Cas9 system delivered by AAV vectors inhibited neovascularization in animal models of laser-induced choroidal neovascularization and oxygen-induced retinopathy upon intravitreal injection 183.

### 7.3. Metabolic diseases

#### Familial hypercholesterolemia

The common genes responsible for more than 90% of familial hypercholesterolemia involve LDLR as the primary gene, alongside APOB and PCSK9. Familial hypercholesterolemia can be divided into two types in accordance with whether only one allele is affected or whether both alleles are affected simultaneously, which are respectively denoted as heterozygous familial hypercholesterolemia or homozygous familial hypercholesterolemia 184. Through the use of CRISPR technology, such as CRISPR/Cas nuclease and CRISPR base editor, *in vivo* modification of individual genomes can be achieved. In animal models with familial hypercholesterolemia variations, RNA-guided CRISPR/Cas9 nucleases repaired LDLR point mutations by disrupting the DNA double strands of targeted genes 167. Therefore, LDLR expression partly recovered and the level of LDL-C decreased. Furthermore, targeting PCSK9 may be ineffective for homozygous familial hypercholesterolemia patients with negligible or absent LDLR function. However, further research on off-target effects and safety in humans is still needed.

#### Phenylketonuria

Phenylketonuria can be caused by disease-causing variations in the phenylalanine hydroxylase (PAH) gene, inducing an increase in blood phenylalanine, which causes neurotoxicity 185. Current drug administration and diet control approaches are not immediately effective and decrease instead of normalizing blood phenylalanine levels 186. The most common PAH variant in phenylketonuria is the P281L variant. Adenine base editing successfully restored the P281L variant in cell lines and humanized phenylketonuria model mice. In addition, after correcting PAH in the livers of humanized phenylketonuria mice, persistent normalization of the blood phenylalanine concentration within 48 hours after intervention was observed. These results illustrate that CRISPR technology holds great potential for treating phenylketonuria, but further exploration is essential.

#### Cystic fibrosis

Cystic fibrosis represents an archetypal inherited disease caused by inactivating mutations in the CFTR gene 187, predominantly influencing the reproductive system, digestive system and respiratory system. Gene therapy targeting cystic fibrosis has been challenging. The lungs are chronically exposed to harsh external environments and are endowed with defense systems to prevent inhaled pathogens and particles from entering lung cells. By utilizing lung-targeting lipid nanoparticles (LNPs) that contain CRISPR components for genome editing, intravenous airway targeting was achieved in the animal model of cystic fibrosis 188. LNPs carrying CRISPR base editor and guide RNA were able to accurately correct the CFTR gene mutations that resulted in cystic fibrosis 187.

### 7.4. Tumor

Rapid progress in CRISPR technology can be leveraged to address multiple challenging and fundamental issues related to malignant tumors. Targeting a range of tumors is currently among the most common clinical applications of CRISPR editing 14. This section focuses on preclinical and clinical studies, including CAR-T and TCR-T based therapeutic research. Multiple CRISPR-based treatments for cancer are currently undergoing clinical trials (Table 5). For example, in one clinical trial, cervical cancer-associated HPV E6/E7 oncogenes were targeted using CRISPR technology (ClinicalTrials.gov Identifier: NCT03057912) 189.

Recent investigations have focused on enhancing CRISPR editing efficacy in immune cells to correct pathogenic mutations 190,191. For example, genome targeting via CRISPR technology can correct disease-causing IL2RA mutations in T cells and partly salvage IL2RA expression 190. With the continuous development of adoptive immune cell therapy, there are also increasing opportunities for exploring novel targeted therapies based on CRISPR-engineered cells. Chimeric antigen receptor T-cell immunotherapy (CAR-T) allows genetically edited T cells from patients to express a chimeric antigen receptor (CAR) on their surface, resulting in sustained antitumor effects (Figure 6A) 192. These engineered CARs help T cells specifically identify and combine with unique antigens on tumor cells, thereby activating antitumor cytotoxicity. CAR-T cell therapy may significantly relieve manifestations in patients with various kinds of blood cancers and solid tumors 193. In addition, immunological therapy associated with gene correction via CRISPR is rapidly moving toward clinical practice. One clinical trial utilized Cas9 nucleases to modify autologous T cells *ex vivo*, knocking out the immune checkpoint PD-1 and endogenous T-cell receptor (TCR) to avoid mismatches or abnormal signaling, facilitating specific edited TCRs that could target tumor antigens 194. The transfer of edited T cells back into patients presents significant prospects for the immunotherapy of malignant tumors (Figure 6B). Nevertheless, these applications are still under continuous development, with sustained efforts to realize efficacious and safe targeted gene therapy tailored to immune cells.

Importantly, the crosstalk between malignant cells and the tumor microenvironment (TME) also regulates tumor development 195-198. CRISPR/Cas methods can precisely target genes that regulate the immune TME and metabolic pathways within tumors, thereby inhibiting tumor growth and metastasis 199. Modulating the TME via CRISPR/Cas technology disrupts the homeostasis that supports tumor cells, thereby suppressing the abnormal behavior of tumors 200,201. In summary, by targeting the genetic basis of tumors, immune cells and TME, CRISPR-based gene editing paves the way for fundamentally altering the behavior of tumors and their interactions with adjacent tissues, revolutionizing cancer therapy.

### 7.5. Duchenne muscular dystrophy

Duchenne muscular dystrophy (DMD) represents a hereditary muscle disorder 202. CRISPR/Cas9 technology works at the DNA level, offering innovative prospects for DMD gene therapy. Furthermore, considerable progress in the field of DMD has been achieved since the emergence of genome editing via CRISPR 63. AAV9 delivery of CRISPR/Cas9 via intraperitoneal, intramuscular, or systemic routes resulted in the expression of corrected dystrophin in animal models of DMD, partly restoring myocardial or skeletal function 202-204. The general‌ approach of these therapies is to correct point mutations in mouse models of DMD 202-204. In addition, *in vitro* genome editing of iPSCs represents a viable strategy for DMD therapy 205. Different types of mutations require different strategies. Exon knock-in by the CRISPR/Cas9 system via iPSC electroporation seems to be a beneficial approach for rescuing DMD with dystrophin gene mutations 205. After iPSCs are modified and differentiated, functional dystrophin can be expressed 205. Similar results were obtained when the reading frames of iPSCs were recovered by CRISPR technology in DMD with frameshift mutations 206.

### 7.6. Hereditary hearing loss

Hereditary hearing loss is a typical illustration of the application of gene therapy in inherited diseases 207. Recently, more than 150 pathogenic genes related to hereditary hearing loss have been identified 208. Owing to the increasing concern about hearing impairment, increasing numbers of studies are endeavoring to treat hereditary hearing loss by gene therapy, which is a significant single-gene disorder and the most prevalent category of congenital deafness. Currently, the prospective curative efficacy of the smallest Cas13 protein, CasRx, has been explored in neonatal Beethoven mice 209. The Tmc1 mRNA level was downregulated by 70% via the CRISPR/CasRx system, and this treatment increased the survival rate of hair cells and alleviated hearing impairment 209. This experimental verification illustrated the effectiveness and safety of CRISPR technology in the management of inherited hearing loss 210.

### 7.7. Neurodegenerative diseases

#### Alzheimer's disease

Causative gene mutations have been found in approximately 1% of familial Alzheimer's disease cases 211. The CRISPR/Cas9 tool has attracted extensive attention considering treatment strategies for Alzheimer's disease involving the correction of genetic defects 212. Utilization of the CRISPR system in Alzheimer's disease has been described, which remains a continuously growing research field 165. Previous research has reported the utilization of dual AAV vector system to target the mutant gene that causes Alzheimer's disease, one packaging APPsw-specific gRNA and the other containing Cas9. The virus was assessed in Tg2576 mice by intrahippocampal injection and was also evaluated in neuronal cells derived from Tg2576 mouse embryos, which exhibited decreased Aβ production 213. Furthermore, brain abnormality and cell death in animal models of Alzheimer's disease were rescued by targeting the methylation of APP through a dCas9–Dnmt3a approach 165.

#### Parkinson's disease

Currently, no strategy has been developed to completely cure Parkinson's disease, but genome editing techniques, particularly CRISPR/Cas9, are expected to achieve perpetual correction in hereditary Parkinson's disease with mutated genes 214. Gordon *et al*. reported that augmenting PK2 signalling exerts a neuroprotective effect, while blocking its receptor accelerates dopaminergic neuron loss in animal models of Parkinson's disease 215. Furthermore, CRISPR/Cas9-mediated knockout of PK2 was shown to exacerbate neuronal susceptibility to neurotoxin-induced cell death, an effect reversed by recombinant PK2 214,215. In addition, Chen *et al*. applied CRISPR system to downregulate SNCA in human embryonic stem cells, slowing the progression of SNCA-related pathological results 216.

#### Huntington's disease

Huntington's disease, an autosomal dominant disorder, arises from CAG repeat expansion in the huntingtin (HTT) gene, leading to the production of mutated HTT protein (mHTT) 211. CRISPR gene editing can selectively inactivate mHTT in patient-derived fibroblasts 217, Huntington's disease model mice 218 and human-differentiated iPSCs 219. It was first used for the permanent and selective inactivation of mHTT alleles in patient-derived fibroblasts by specialized allele-specific CRISPR/Cas9 to remove a broad region of HTT DNA, leading to the almost complete elimination of mRNA and mHTT proteins 217. In addition, Kolli *et al*. studied the inhibitory effects of CRISPR/Cas9 systems on mHTT. The results demonstrated that mHTT silencing by CRISPR technology reduced the expression of mHTT in mesenchymal cells 220.

### 7.8. Autoimmune diseases

#### Systemic lupus erythematosus

Systemic lupus erythematosus can be featured by the destruction of self-antigen tolerance and manifests as the production of autoantibodies, inflammation and organ damage 221. Although the pathogenesis of systemic lupus erythematosus has not yet been fully clarified, genetic traits constitute the etiology of this disorder 222. The CXorf21 gene has been proposed as a suitable candidate gene for CRISPR/Cas9 treatment in systemic lupus erythematosus. The CXorf21 knockdown *in vitro* by CRISPR/Cas9 led to lower expression levels of IL-6 and TNF-alpha 168.

#### Type 1 diabetes

Type 1 diabetes can result in damage to pancreatic β cells, which mediate insulin secretion. Transplantation of insulin-secreting β cells may be an efficient and definitive treatment for type 1 diabetes 223. Although the supply of primary β cells is incredibly limited, methods for generating β cells from pluripotent stem cells are being widely studied. Pluripotent stem cells can undergo gene editing through CRISPR/Cas to correct mutations that cause diabetes and eventually obtain effective β cells 224. For instance, CRISPR technology was utilized to correct mutations of INS genes or WFS1 genes in iPSCs obtained from diabetes patients. Corrected iPSCs subsequently differentiated into stem cell-derived β cells with normal insulin folding and function, demonstrating considerable value for clinical application 225,226.

### 7.9. Infectious diseases

Infectious diseases are a serious challenge for public health and require new strategies for treatment 227. CRISPR-based gene therapy targeting pathogen-specific genes provides a new strategy to fight infectious agents. CRISPR can also be used to identify the key host properties that pathogens exploit in infectious diseases, facilitating the development of creative approaches to reduce their virulence 228. In addition, CRISPR can be used to develop diagnostic methods that contribute to the prompt detection of distinctive genetic markers of pathogens. One clinical trial (ClinicalTrials.gov identifier: NCT03164135) demonstrated that CRISPR gene editing targeting CCR5 could suppress human immunodeficiency virus type 1 (HIV-1) 229. Furthermore, viral genomes may be removed from human cells by CRISPR 230. One study using eight different sgRNAs that target hepatitis B virus revealed that integration of the viral genome into hepatocellular carcinoma cells was suppressed by CRISPR technology, consequently decreasing the expression of viral antigens.

## 8. Progress in delivery systems

Multiple methods for delivering CRISPR/Cas components to promote optimal gene editing activity have been explored 2. CRISPR systems can be delivered primarily via physical, viral and non-viral approaches. Each delivery platform exhibits unique strengths and inherent limitations that must be carefully evaluated depending on the specific therapeutic application 231. Physical methods, notably microinjection and electroporation, are particularly well-suited for *in vitro* and* ex vivo* applications due to their high efficiency and lack of cargo size constraints, but they are unsuitable for systemic *in vivo* delivery 20. Viral vectors, most notably AAV, remain unparalleled in terms of efficiency and tissue-specific tropism, but they are associated with several concerns, including immunogenicity and limited cargo capacity 232-234. Non-viral delivery approaches, particularly LNPs and emerging platforms such as engineered exosomes and virus-like particles, are progressively narrowing the efficiency gap while offering superior safety and targeting specificity 235-238. The convergence of these technologies exhibits considerable potential to broaden the applications of CRISPR system.

### 8.1. Physical delivery methods

Physical delivery methods can deliver CRISPR components by penetrating cellular barriers 20,28. The predominant physical delivery approaches include microinjection, electroporation, and hydrodynamic delivery. Microinjection involves a needle (0.5–5.0 μm in diameter) to deliver small quantities of substances into specific intracellular regions, such as the cytoplasm or nucleus 28. Additionally, unlike size-limited delivery vectors, microinjection is not constrained by cargo size 20. Moreover, microinjection is most appropriate for *in vitro* as well as *ex vivo* applications. As a visually observable and real-time traceable method, it is widely applied in fertilized mouse eggs and zebrafish embryos because of its low lethality and high delivery efficiency 239. However, the primary limitations of microinjection are its manual operation and incompatibility with *in vivo* applications 20.

Compared with other delivery methods, electroporation is less cell type dependent and highly efficient at transfecting traditionally refractory cells. Similar to microinjection, electroporation remains effective for *ex vivo* or *in vitro* applications. *Ex vivo* electroporation has been extensively utilized in clinical trials, most notably in the realms of cancer immunotherapy and the treatment of inherited hematological disorders 11,26,194. However, electroporation for CRISPR delivery has challenges, such as cytotoxicity, high costs, and operational complexity 240.

The efficiency of hydrodynamic delivery depends primarily on the anatomical structure of the target organ and its expansion rate post-intravascular injection 241. When a substantial amount of solution is abruptly infused into the capillaries, it causes rapid expansion of the cell membrane. This leads to invagination formation, through which the solution gains access to the cellular interior without exogenous delivery vehicles 242. This method is commonly used in gene therapy research for rodents. However, it is not considered suitable for application in large animals or humans, primarily due to its impracticality and insufficient safety profile 241. For example, acute overload of the systemic circulation may lead to severe circulatory dysfunction 243.

Taken together, physical delivery methods, notably microinjection and electroporation, are particularly well suited for *in vitro* and* ex vivo* applications. Nevertheless, their insufficient safety profile and operational complexity limit their utility for systemic *in vivo* delivery, highlighting the need for complementary viral or non-viral strategies when tissue-specific editing is required.

### 8.2. Viral delivery methods

Viral delivery approaches have been identified as the most widely utilized strategies for efficiently delivering CRISPR components* in vivo* 77. High-capacity viral vectors, including lentivirus (LV) and adenovirus (AdV) vectors, can encapsulate every component of the CRISPR system 77. Nevertheless, AAV vectors with favorable efficiency have received much attention (Table 6). In addition, different organs can be targeted by specific AAV serotypes 233. For example, AAV5 was utilized to deliver CRISPR system for treating Leber congenital amaurosis 10 24. The advantages of AAV include high transduction efficiency, low carcinogenic risk, serotype-related target cell specificity, long-lasting therapeutic effects, and remarkable clinical success 244-246. CRISPR system delivery via AAV has performed well, but AAV can encapsulate only a limited amount of cargo 234. Therefore, compared with relatively large SpCas9 (4.2 kb), smaller Cas9 orthologs might be more appropriate for delivery by AAV 24. Although dual AAV vectors can be utilized, an all-in-one construct with a truncated Cas9 nickase represents a more appealing solution that has recently been investigated 247. Moreover, although the efficiency of the virus vector deserves serious consideration, safety issues should not be ignored. In addition to the limited AAV package size, DNA encapsulated in AAV may unintentionally be integrated into the induced on-target DSB 248. Because of the AAV vector DNA and AAV capsid, gene therapy delivered by AAV has the risk of activating the host immune system 233. For this reason, many studies have focused on regulating AAV-related immunogenicity 249. For example, optimized AAV serotypes with increased efficiency at low doses can partially overcome these challenges 250.

### 8.3. Non-viral delivery methods

Effective and safe non-viral delivery strategies for gene editing are urgently needed and have unique benefits in delivering gene cargo, including enabling instantaneous expression, low immunogenicity, fewer cargo size restrictions, and convenient mass production 268. Many biomaterials, such as inorganic nanoparticles (NPs), polymeric NPs, LNPs, metal–organic frameworks, DNA origami, engineered exosomes, and virus-like particles, exhibit a significant ability to deliver CRISPR components for robust gene editing 2,269,270.

#### 8.3.1. Inorganic NPs

##### Gold nanoparticles

Gold nanoparticles (AuNPs), which exhibit distinct physical and chemical characteristics, are considered excellent non-viral vectors 271. The physicochemical characteristics of AuNPs enable regulation of the surface charge by combining with cationic molecules to facilitate DNA binding via electrostatic interactions 272. The effectiveness of AuNPs as non-viral delivery vectors has been illustrated in numerous studies 273,274. Furthermore, arginine-modified AuNPs were shown to deliver RNPs directly to the cytoplasm via membrane fusion, resulting in high genome editing efficiency 275,276. A complex comprising the Cas effector, AuNPs and an endosomal destructive polymer (CRISPR-Gold) was effectively used for the treatment of fragile X syndrome and DMD in mouse models 277,278. Even after repeated injections, no obvious toxicity was detected in this treatment. Recently, the CRISPR system was reported to be delivered by colloidal AuNPs into progenitors *in vitro* and effectively perform genome editing, providing a promising therapeutic approach for various diseases 279. Furthermore, AuNPs with photothermal features can be extensively used for photothermal therapy or stimulating the release of cargo 280,281, demonstrating promising prospects for CRISPR-based gene therapy delivered by AuNPs. Accordingly, CRISPR/Cas9 can be delivered via an integrated‌ approach in which AuNPs and lipids are combined to target the Plk-1 gene to suppress melanoma 67. Moreover, as illustrated in Figure 7A, which depicts the synthesis of Cas9-loaded gold nanorods and the combined photothermal/gene therapy process, synergistic photothermal/gene therapy demonstrated significant antitumor efficacy through effective delivery of CRISPR components and gold nanorods 282.

##### Black phosphorus nanosheets

Black phosphorus nanosheets have a two-dimensional structure and have sparked intense research focus because of their unique physical and chemical characteristics 283,284. The periodic atomic grooves on the surfaces of black phosphorus nanosheets offer appropriate anchoring sites with great prospects for the loading and delivery of biomolecules 284, including CRISPR/Cas9 RNP. Black phosphorus nanosheets loaded with Cas9 RNPs were effectively constructed and engineered with three nuclear localization signals (NLSs) at the C terminus (Cas9N3). As depicted in Figure 7B, which illustrates the design of black phosphorus nanosheets for delivering CRISPR components and related intracellular delivery pathways, Cas9N3–black phosphorus nanosheets entered cells efficiently, subsequently triggering endosomal escape upon the biodegradation of black phosphorus nanosheets and cytosolic release of Cas9N3 complexes, thereby achieving robust genome editing 284. Furthermore, black phosphorus nanosheets for delivering Cas13a/crRNA complexes were developed to suppress Mcl-1 transcription levels for the treatment of breast cancer 285.

##### Upconversion nanoparticles

Upconversion nanoparticles can release biomacromolecules with high spatiotemporal precision via photoregulation, which can transform long-wave light into short-wave light 286. When stimulated by 980 nm near-infrared light, upconversion nanoparticles emit 409 nm light to trigger protoporphyrin to generate ^1^O_2_, which has enhanced tumor suppressive effects resulting from the reduced expression level of Nrf2 287. Robust gene editing and effective photodynamic therapy were subsequently accomplished. Upconversion nanoparticles have become a research focus in the area of inorganic nanomaterials on account of their superior characteristics, such as safety and reduced side effects of near-infrared triggered therapy 288. As shown in Figure 7C, one study reported a multifunctional DNA/upconversion nanoparticle complex that was able to co-deliver protoporphyrin, hemin and CRISPR/Cas9 to achieve combined photodynamic therapy 287. Upconversion nanoparticles can convert near-infrared irradiation into short-wavelength irradiation and activate protoporphyrin to convert O_2_ into cytotoxic ^1^O_2_ via the CRISPR/Cas9-mediated downregulation of Nrf2, which inhibits the elimination of ^1^O_2_. The effective accumulation of cytotoxic ^1^O_2_ within tumor cells promotes apoptosis and effectively inhibits tumor growth 287.

#### 8.3.2. Polymeric NPs

Highly selective and specific delivery is critical and can greatly extend the utilization of CRISPR gene editing. Polymeric NPs have shown particular promise in preclinical studies. A major class of compounds in polymeric NPs are cationic polymers, including poly(β-amino esters) (PBAE) and poly(ethyleneimine) (PEI), which contain positively charged groups that can interact electrostatically with nucleic acids 289. By using polymeric NPs, CRISPR/Cas components can be delivered to specific cell types in a targeted manner. Through electrostatic interactions between the cationic polymer and the cargo itself, the CRISPR cargo can be condensed into a solid nanocomplex. The amphiphilic polymer subsequently encapsulates the complex to form multilayer NPs, thereby enhancing CRISPR delivery. In addition, alpha-helical cationic peptides may be used as both cell-penetrating agents and effective gene carriers 290. In general, the excellent affinity between the active targeting portion and cell surface receptors can contribute to the efficacy of targeted delivery systems. For example, the CD44 receptor can bind specifically to hyaluronic acid (HA). Accordingly, the protein-based drug ribonuclease A was modified with HA and lipid molecules to increase the targeting and hence killing of tumor cells relative to that of the ribonuclease itself 238.

Recently, the combination of sgRNA and Cas9 RNP (nanoRNP) was shown to be delivered by polyethylene glycol (PEG)-based NPs, offering a viable approach for efficiently overcoming tumor heterogeneity 291. In this system, the expression of runt-related transcription factor 1 and signal transducer and transcriptional activator 3 were inhibited *in vivo* by nanoRNPs, which effectively inhibited tumor progression. Moreover, compared with commercially available reagents, core‒shell polymeric NPs presented increased transfection efficiency with minimal side effects 292. Besides, researchers have used polymer NPs to deliver a CRISPR/Cas13a system targeting PD-L1 to activate antitumor immunity mediated by T cells, restoring an immunosuppressive TME 293. As illustrated in Figure 7D, which presents the preparation of PGBA-RNP polymeric NPs and an overview of mechanisms for genome editing, PGBA NPs enabled efficient RNP delivery for *in vivo* genome editing, resulting in a marked reduction in PCSK9 levels 294. One nano-CRISPR scaffold with a versatile copolymer was designed for the co-delivery of CRISPR/dCas9 and cisplatin 295. Furthermore, the nano-CRISPR scaffold inhibited the progression of malignant melanoma, resulting in strong tumor-suppressive immunity 295.

Therefore, the delivery of CRISPR cargo by NPs can overcome biosafety issues related to viral delivery approaches and improve the diversity of non-viral vectors, which may provide effective platforms for CRISPR-based disease therapies 292.

#### 8.3.3. LNPs

LNPs have been widely used and are currently accepted as favorable carriers for CRISPR delivery. The widely used LNPs comprise four components: PEG lipids, ionizable lipids, cholesterol, and helper lipids 289. Every component of the LNPs influences delivery through a distinct mechanism. Customizing the surface properties and composition of LNPs can markedly boost delivery performance 296. Critical factors for enhancing delivery efficiency include optimizing the size of encapsulated proteins, the overall diameter of LNPs, and the surface charge 296,297. Besides, LNPs with flexible lipid shells enter cells less effectively compared to LNPs with rigid shells 298. LNP-encapsulated Cas9/sgRNA complexes were used to mediate CRISPR editing of transthyretin in humans (NTLA-2001), resulting in a substantial decrease in serum transthyretin levels and validating the therapeutic potential of optimized hepatic delivery 299.

A principal limitation of intravenously administered LNPs is their natural tropism for the liver 171, which restricts extrahepatic CRISPR applications 300. Nevertheless, this tropism can be modulated by altering lipid molar ratios and administration routes, or by conjugating target-specific ligands 171,301. High-throughput molecular barcoding technology was used in conjunction with traditional screening methods to assess LNP formulations concurrently for the biodistribution and efficiency of gene editing. This approach led to the exploration of lung-tropic LNPs that demonstrated effective gene editing in pulmonary epithelial and endothelial cells (as shown in Figure 7E, which depicts CRISPR/Cas9 RNP-encapsulated LNPs for *in vivo* delivery), establishing an extremely selective non-hepatic delivery method for lung-specific editing applications 235. Similarly, LNPs loaded with Cas9 mRNA originally targeting the liver were redirected to the spleen after being formulated with anionic lipids 302. In head and neck cancer, intratumoral delivery of EGFR-targeted CRISPR-LNP functionalized with targeting moieties enabled safe and efficient SOX2 knockout, demonstrating the potential of treating solid tumors and inducing durable therapeutic responses 303. In addition, SM102-based LNPs co-delivering SpCas9 mRNA and sgRNA enabled efficient Mgp knockout in trabecular meshwork cells, demonstrating their potential for targeted gene editing 304. A recent organic solvent-free LNP formulation that avoided cholesterol further reduced hepatic accumulation and immune activation while delivering multiple sgRNAs and Cas9 mRNA, demonstrating the feasibility of extrahepatic CRISPR applications 305. Collectively, LNPs represent the most widely utilized non-viral delivery approach in biotherapy, and their capacity to facilitate CRISPR delivery holds significant value for clinical applications.

#### 8.3.4. Metal–organic frameworks

Metal–organic frameworks are crucial types of gene delivery vehicles owing to their distinctive characteristics, such as tunable pore structure, biodegradability and drug-loading capacities 306. Metal–organic frameworks feature highly tunable pore structures and a large specific surface area, enabling efficient loading of biomolecules such as sgRNA and Cas proteins 307,308. In addition, metal–organic frameworks have good chemical stability and biocompatibility, exhibiting low immunogenicity and low toxicity 309. Surface functional modification of metal–organic frameworks (for instance, folic acid modification) can enhance targeting capability and enable spatiotemporally controlled release of cargo via photothermal effect or ultrasonic treatment 310,311. For example, nano-sonosensitizer metal–organic frameworks, termed P/M@CasMTH1, can deliver CRISPR component for targeting MTH1 and be utilized as a sonosensitizer to specifically trigger genome editing, resulting in antitumor effects (as illustrated in Figure 7F for the preparation of P/M@CasMTH1 and ultrasound-triggered gene editing-augmented sonodynamic therapy) 312. Moreover, biocompatible nano-sized zeolitic imidazolate framework-8 (ZIF-8) can be utilized to deliver organic small molecules and bioactive macromolecules 313-315. Furthermore, considering the simplicity and cost-effectiveness of the preparation process 316,317, ZIF-8 is highly suitable for controlled RNP delivery, thereby achieving effective genome editing. Alsaiari *et al*. illustrated that metal–organic framework ZIF-8 might function as a superior non-viral CRISPR/Cas9 delivery system that is biocompatible and has a controllable CRISPR/Cas9 component delivery capability 318.

#### 8.3.5. DNA origami

As a burgeoning field of DNA technology, DNA origami folding ssDNA (known as scaffold strands) with a thousand bases long and many synthetic oligonucleotides (referred to as staple strands) has emerged as an excellent design strategy, primarily because the use of scaffold strands produced by enzymatic means reduces self-assembly errors and production costs 319-323. DNA origami enables the strategic design of DNA nanostructures with appropriate sizes and specific geometric shapes 324-326. With these advantages, structurally diverse DNA origami is applicable for delivering Cas9 and Cas12a RNPs 327,328. As depicted in Figure 7G, a CRISPR system based on DNA origami was constructed for effective gene therapy *in vivo* 329. DNA origami loaded with sgRNA/Cas9 complexes was rolled up via locking strands with a disulfide bond. After glutathione reduction, the opened DNA origami delivered the sgRNA/Cas9 complexes through RNase H cleavage to accomplish significant editing of disease-related genes for *in vivo* gene therapy. This gene editing system on the basis of DNA origami offers a favorable approach for advancing gene therapy 329. Although controllable CRISPR/Cas9 systems have been developed using DNA origami 330, DNA origami nanoscaffolds driven by the pathophysiological environment for delivering Cas9 RNPs remain underutilized.

#### 8.3.6. Engineered exosomes

Extracellular vesicles have become promising nanocarriers for bioactive molecules over the past decade 331. Specifically, exosomes represent a subgroup of extracellular vesicles secreted by diverse cell types 236,332, and exhibit unique advantages, including low immunogenicity, superior cargo loading efficiency, reliable stability, favorable biocompatibility, and the capacity to traverse biological barriers 333-337. Consequently, exosomes have attracted widespread attention for drug and gene delivery 338,339. Recently, engineered exosomes incorporating a photoactivatable cargo-release system (MAPLEX) were constructed for epigenome editing *in vivo* 236. The MAPLEX system facilitated the delivery of sgBace1-dCas9-D3A in mice, which resulted in CpG methylation, decreased Bace1 expression, and ameliorated recognition memory deficits and amyloid pathology 236. Furthermore, an exosome-based platform (EMT-Cas12a) was constructed to deliver Cas12a mRNA and crRNAs, resulting in the suppression of HIV while maintaining favorable biosafety 340. For musculoskeletal disorders, chondrocyte affinity peptide-modified MSC-derived exosomes served as biocompatible carriers for CRISPR/Cas9-mediated ASPN knockout in osteoarthritis-affected chondrocytes, demonstrating significant therapeutic efficacy in osteoarthritis models and underscoring the potential of exosome-based platforms for precision gene-targeted interventions 341. Collectively, these studies highlight the versatility of engineered exosomes as programmable delivery vehicles for CRISPR systems across a wide range of disease models. Further improvements in targeting precision and large-scale production will be critical for translating these platforms into clinical gene therapies.

#### 8.3.7. Virus-like particle

The appearance of virus-like particles resembles that of native viruses, but they lack viral genomes and therefore cannot replicate 342. Therefore, virus-like particles can be classified as a non-viral delivery technology. Virus-like particles originate from existing viral scaffolds and make use of the inherent characteristics of viruses to facilitate effective intracellular delivery, such as the capacity to encapsulate cargo, to target distinct cell types, and to escape endosomes 252. Owing to the ability for the transient CRISPR delivery, virus-like particles have received extensive attention with respect to gene therapy 343. As illustrated in Figure 7H, peptidyl virus-like particles that imitate simian virus 40 and human immunodeficiency virus have been constructed 237. Two viral peptides with good targeting ability were able to co-assemble to form biodegradable peptidyl virus-like particles by encapsulating the CRISPR component. The peptidyl virus-like particles successfully crossed the cell membrane and transferred cargo to the nucleus via the viral entry pathway. This study illustrates the significant potential of engineered virus-like particles with desirable properties 237.

## 9. Current challenges in CRISPR technology

Many obstacles remain to be overcome in the development of diagnostic methods and treatments by CRISPR technology 344. Despite the advantages of CRISPR technology, challenges associated with its widespread application persist. Further research can focus on increasing efficacy, reducing off-target effects, optimizing cargo delivery methods, improving safety, and addressing ethical issues.

### 9.1. Efficacy of CRISPR technology

Multiple aspects influencing the efficacy of CRISPR technology are relevant to its wider application. The requirement for a PAM adjacent to the target site restricts the range of targetable disease-causing mutations, which is a key problem limiting efficacy. Research focused on key molecules of the CRISPR system could be beneficial for mitigating this restriction. For example, the novel CRISPR effectors NG-SpCas9 345 and xCas9 346 are able to target DNA sequences using unconventional ‘NG’ PAM sequences rather than the traditional ‘NGG’ PAM sequence. In addition, the RNA-targeting CRISPR/Cas13 system may function as an efficient and safe strategy to accurately modulate the transcripts of disease-causing genes at the mRNA level, avoiding the long-term dangers of modifying genomic DNA 344. Furthermore, the CRISPR delivery systems require further optimization to ensure effective genome editing without unnecessary damage to healthy tissues, which should also decrease costs and improve accessibility 347.

Furthermore, a more fundamental obstacle is the poor predictive validity of current preclinical models for human disease 348,349. Before advancing to clinical trials, CRISPR-based therapies must demonstrate robust efficacy in preclinical models 63. However, interspecies differences in preexisting immunity, target tissue accessibility and cellular uptake frequently result in substantially lower editing efficiency in humans than in murine or other small animals 350,351. Conventional mouse models often fail to recapitulate the genetic background, disease progression, and immunological landscape of human conditions 352. Non-human primate models, although physiologically more similar to humans, are constrained by high costs, ethical concerns, and small sample sizes, precluding large-scale systematic efficacy evaluation 353,354. Even emerging patient-derived organoids remain limited by several inherent limitations, most notably insufficient maturation and high heterogeneity 355. This preclinical-to-clinical translation gap profoundly compromises dose selection, endpoint determination, and risk assessment in trial design 63,356. Overcoming this barrier requires the development of more predictive preclinical models, including humanized animal models, to improve efficacy prediction and reduce the rate of clinical trial failures for CRISPR-based therapies 349,357,358.

### 9.2. Off-target effects

Medical applications require versatility but also rigorous precision to guarantee biosafety. Therefore, many studies have investigated the underlying problem of unwanted genome editing at other gene loci, known as off-target effects 359, which can result in health problems and even promote cancer progression. Therefore, preventing off-target effects is another vital issue to be solved (Figure 8) 34. Off-target effects can be minimized through appropriate control and the use of multiple sgRNAs. Specifically, sgRNAs with fewer nucleotides may exhibit reduced off-target activity 360. In addition to guiding RNA modification, improving the expression of CRISPR effector proteins may also be a suitable strategy for avoiding off-target effects 361. Further studies have also indicated that highly specific Cas9 proteins such as HF-Cas9 361 and eSpCas9 362 are beneficial for regulating protein‒DNA interactions, preventing the mismatch of CRISPR effectors 363. In addition, the retention time and concentration of the Cas protein in cells may be limited to enhance target specificity and avoid off-target effects 24,364,365. Introducing a requirement for the specific activation of Cas9 by exogenous stimuli is another approach 366,367. Moreover, Cas12a, as a high-precision CRISPR effector, has fewer off-target effects than Cas9 344. Suitable carriers for CRISPR components, such as functional nanoparticles, may also maximize the stability of the cargo, improving target specificity 292.

### 9.3. Delivery methods

Safe and efficient delivery of CRISPR/Cas components to target tissues *in vivo* represents another major challenge for clinical translation 368. Specifically, programmable nucleases should be delivered in a transient manner at controlled doses to reduce off-target editing and immunogenicity while achieving sufficient therapeutic benefit 368. CRISPR delivery methods have both advantages and limitations, which can be compared across three key dimensions. With respect to immunogenicity, AAV vectors carry a high risk of preexisting immunity and may elicit capsid-specific T cell responses 369,370. LNPs exhibit low immunogenicity, with innate immune activation induced primarily by lipids that may be mitigated through formulation optimization 371,372. Virus-like particles are considered safer owing to the absence of viral genetic material 370,373,374. Regarding maximum tolerated dose, AAV administration at doses equal to or exceeding 5×10¹³ vg/kg has been associated with toxicity including thrombotic microangiopathies 375. Hepatic accumulation represents the main factor limiting the maximum tolerated dose of LNPs, although NTLA-2001 was generally well-tolerated at 0.1–1.0 mg/kg 299,376. Early preclinical data suggest that virus-like particles may offer increased maximum tolerated dose due to their biodegradability and reduced systemic inflammation 373,374. Considering the feasibility of repeated administration, AAV vectors generally preclude repeated administration because of neutralizing antibodies 372. Accordingly, EDIT-101 was designed as a single-administration therapy. In contrast, LNP-delivered CRISPR therapies have demonstrated the feasibility of repeated dosing, as their low immunogenicity permits effective booster administration 171,372. Virus-like particles may still face constraints for repeated dosing owing to anti-vector immunity, although this is generally more modest than that associated with AAV 377.

Two pioneering *in vivo* CRISPR therapies illustrate how the delivery system fundamentally dictates clinical trial design 180,299. NTLA-2001 employs LNPs to deliver Cas9 mRNA with liver tropism for the treatment of transthyretin amyloidosis, a design that maximizes therapeutic safety 299. Conversely, EDIT-101 relies on an AAV5 vector to deliver the CRISPR components for the treatment of Leber congenital amaurosis 10, depending on sustained long-term expression to achieve durable therapeutic effects after a single dose 180. This contrast demonstrates that the choice of delivery vector determines not only the pharmacokinetic profile of the editing components but also the entire therapeutic regimen, including dosing frequency, long-term safety monitoring, and patient follow-up strategies. These differences underscore the need to develop suitable delivery systems that combine specific targeting, robust transfection, superior cargo loading efficiency, and minimal immunogenicity. Furthermore, the long-term biosafety and minimal toxic effects of delivery vehicles must be rigorously evaluated before advancing CRISPR-based therapies into clinical practice 20.

### 9.4. Safety and ethical issues

While CRISPR faces numerous challenges related to its utilization, safety and ethical issues of genomic editing seem to present the greatest challenge. In particular, CRISPR/Cas may cause severe damage to tissues and result in systemic side effects‌ due to host immunogenicity and genome integration. One major concern for safety that needs to be addressed is the immunogenicity of the Cas protein itself or its delivery components 35. CRISPR therapies that require the sustained expression of Cas9 need to overcome preexisting immunity 378. Therefore, removing antigen regions of the CRISPR system or designing Cas9 variants to avoid universal‌ly immunogenic epitopes may be effective strategies 378. With the great progress in CRISPR technology, ethical issues have become a primary consideration for clinical applications 379. Investigating the effects of genome editing on nontarget organisms is also essential, as these effects may persist and expand‌ with each generation, leading to disastrous‌ effects‌ on the balance of ecosystems 380. Another key concern is the possibility of modifying human germ cells. To date, genome editing experiments have been primarily conducted on somatic cells, and alterations to the germline genome in a zygote or embryo is widely considered unacceptable because genome editing could introduce mutations with unpredictable adverse reactions in future generations 381.

## 10. Conclusions and perspectives

The advancement of CRISPR technology is mainly driven by the continuous optimization of Cas nucleases. High-fidelity variants such as HF-Cas9 and eSpCas9 markedly improve specificity and minimize off-target effects. Compact Cas enzymes, including Cas12f variants, represent a significant breakthrough in overcoming AAV packaging limitations. Furthermore, chemical and physical control strategies have expanded the spatiotemporal precision of CRISPR systems. Precise editing strategies, particularly base editing and prime editing, have achieved unprecedented accuracy in genomic modifications. Apart from genome editing, CRISPR technology can also be utilized for transcriptional activation and inhibition, such as CRISPRa and CRISPRi systems. Collectively, with the constant progress of CRISPR technology, genome editing has become highly useful in fundamental biological research. Applications ranging from CRISPR screening to disease model construction have accelerated not only basic research but also innovation across the life sciences. Furthermore, CRISPR technology has demonstrated remarkable prospects for diverse diseases, including hematological, eye-related, metabolic, neurodegenerative, and autoimmune diseases, as well as other intractable disorders. Ongoing preclinical and clinical trials will help determine the full capabilities of CRISPR.

Despite the considerable capabilities of CRISPR technology, challenges persist in terms of its widespread application. Delivery remains a major bottleneck, with inherent trade-offs between viral and non-viral systems. Viral vectors offer high transfection efficiency but are limited by restricted cargo capacity, whereas non-viral systems exhibit improved safety profiles but display substantially lower efficacy. In terms of viral vectors, dual-AAV system has emerged as an effective strategy to circumvent packaging constraints. Parallel advancements in non-viral approaches include stimulus-responsive polymers enabling microenvironment-dependent payload release, LNP optimization by modifying the surface charge, and virus-like particles with superior targeting specificity. Rapid progress in delivery systems not only facilitates fundamental research but also promotes the clinical application of CRISPR technology.

Beyond delivery, off-target effects remain a critical concern even with high-fidelity Cas variants, particularly for systemic *in vivo* applications. Notably, rapidly developing artificial intelligence (AI) techniques, such as machine learning and deep learning, address these challenges by leveraging large-scale experimental datasets to improve gRNA design, predict off-target activity, and increase editing efficiency 382,383. Trained on comprehensive datasets, machine learning algorithms are able to predict gRNA activity and off-target risks, optimizing gRNA design in terms of both efficiency and precision 382. Moreover, AI has also facilitated the development of engineered editing proteins. Deep learning tools for predicting protein structure, including AlphaFold 384-386 and RoseTTAFold 387, provide widespread access to high-resolution structural information for Cas nucleases and other genome editing enzymes. The integration of AI and CRISPR technology provides novel approaches for personalized gene therapies.

Collectively, ongoing advancements in CRISPR technology can not only optimize therapeutic efficacy and precision but also surmount the current limitations. Multiple preclinical studies and clinical trials worldwide are significantly expanding the therapeutic prospects of CRISPR technology. These investigations have demonstrated the effectiveness and safety of CRISPR technology in disease treatment. With continuous refinement, CRISPR technology holds immense potential to achieve unprecedented breakthroughs in biomedical research and alleviate previously intractable diseases.

## Figures and Tables

**Figure 1:**
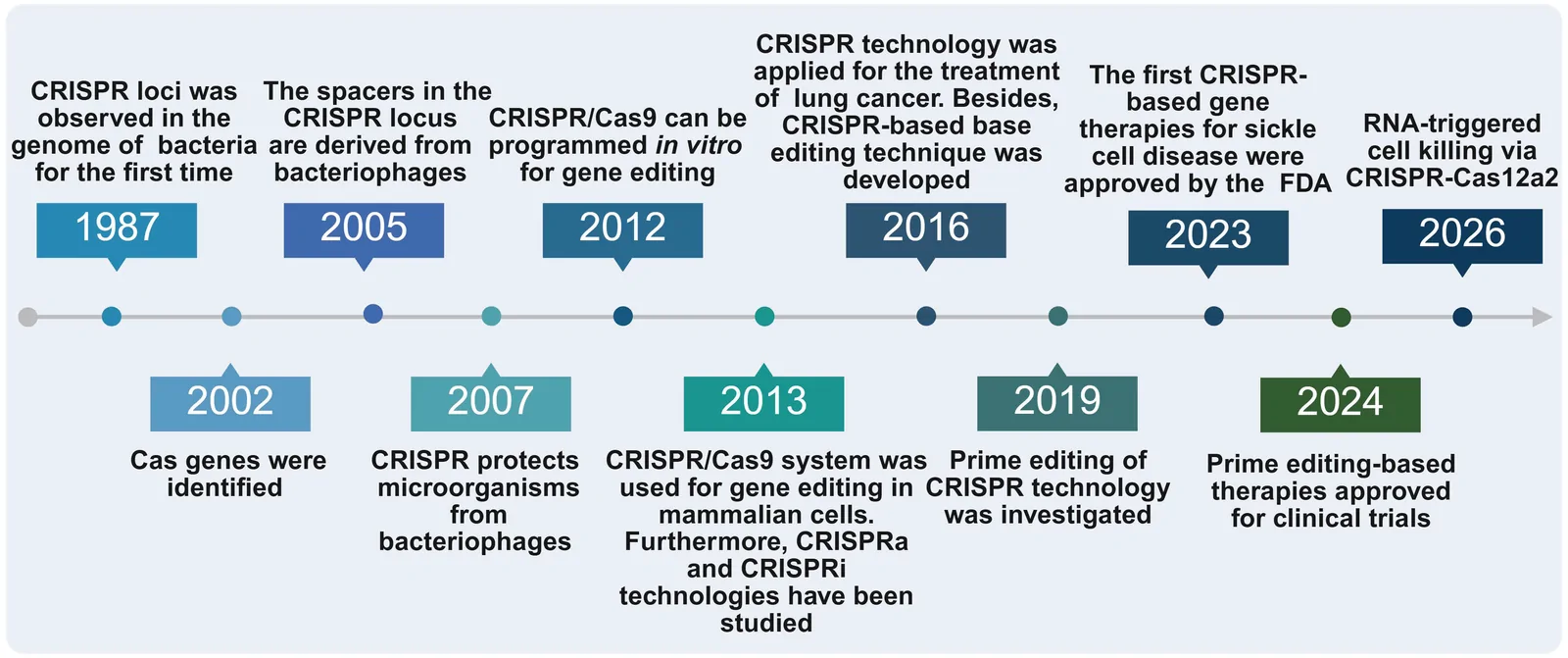
** Brief timeline for the development of CRISPR technology 3-16.** Created with BioRender.com.

**Figure 2:**
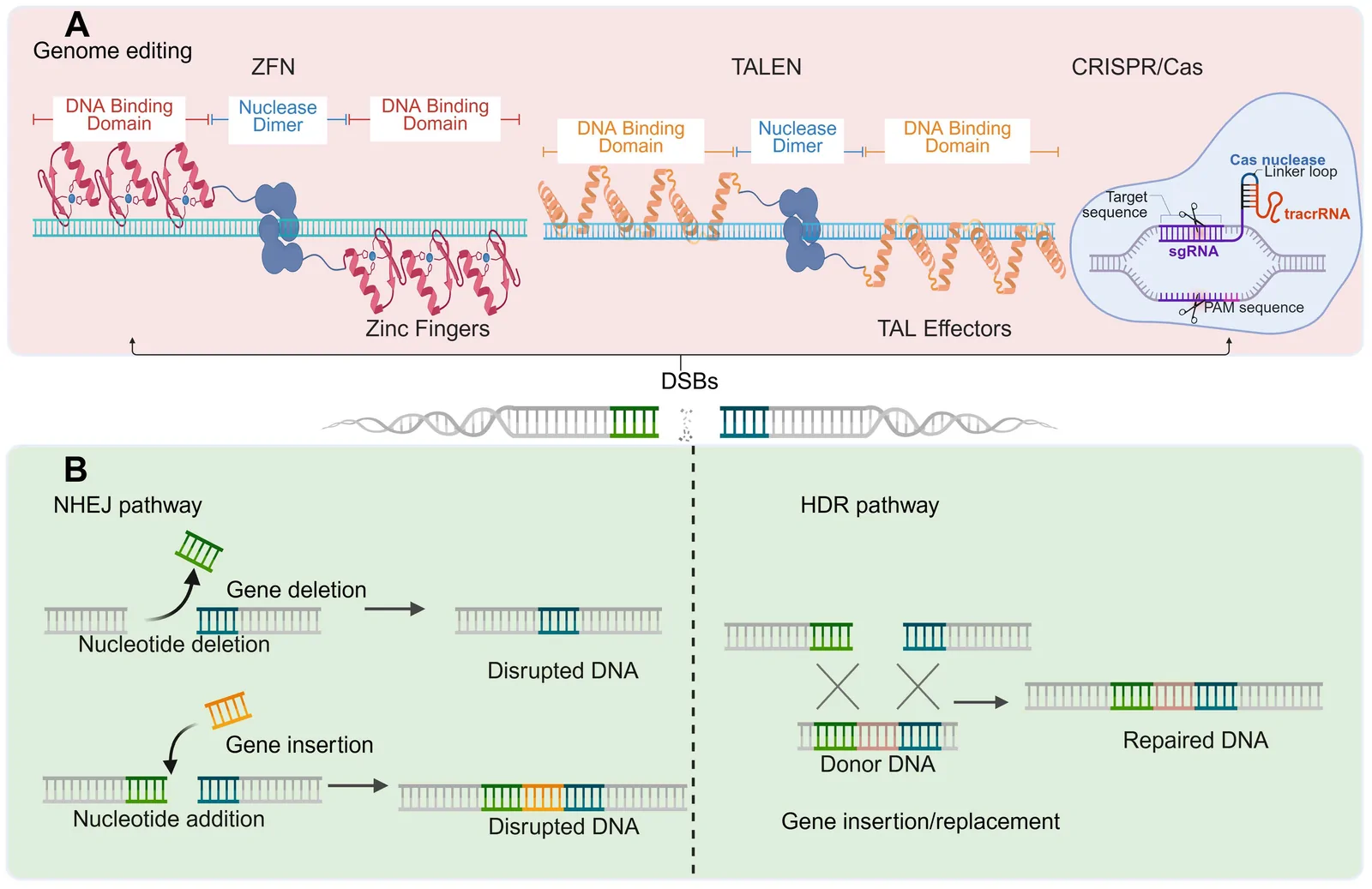
** DNA repair mechanisms for genome editing. (A)** ZFN, TALEN, and CRISPR/Cas technologies can induce DSBs at targeted sites. **(B)** Endogenous DNA repair can be completed via the NHEJ or HDR pathway. Created with BioRender.com.

**Figure 3:**
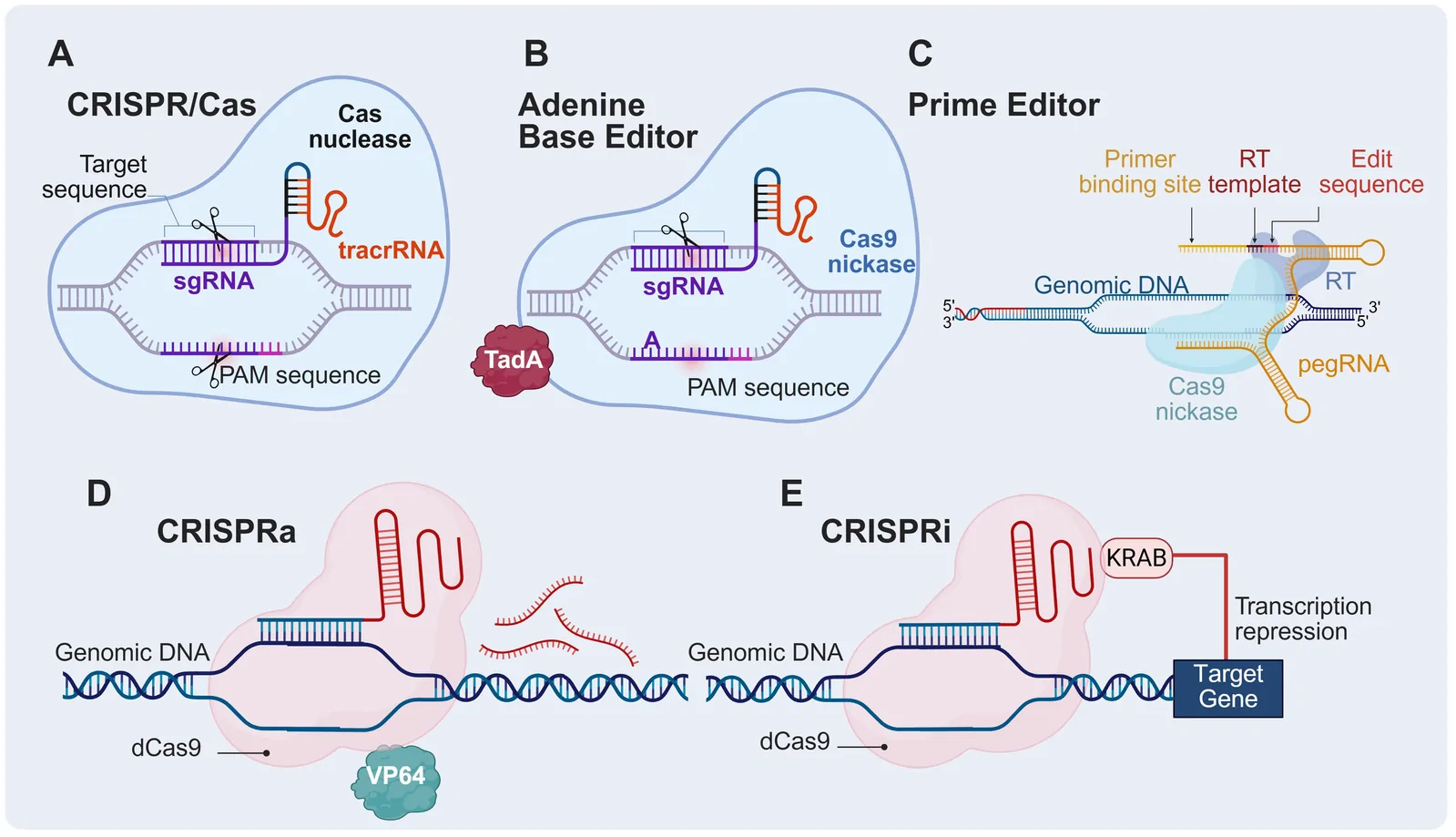
** Extended CRISPR technology. (A)** Conventional CRISPR/Cas system: The sgRNA directs the Cas nuclease to the target genomic locus adjacent to the protospacer adjacent motif sequence. The Cas nuclease introduces DSBs at the target site, which trigger DNA repair mechanisms such as NHEJ or HDR. **(B)** Adenine base editor: The nCas9 is fused to an adenine deaminase domain (e.g., TadA). This system mediates precise single-base transition at the target site without generating DSBs. **(C)** Prime editor: The system generally exploits nCas9 fused to reverse transcriptase and utilizes pegRNA. Specifically, pegRNAs comprise sgRNA scaffold with a 3' extension that provides a reverse transcription template, thereby creating specific alterations at the target site. **(D)** CRISPRa: A catalytically inactive dCas9 is fused to transcriptional activator domains (e.g., VP64). The complex is recruited to the promoter region of the target gene to upregulate endogenous gene expression at the transcriptional level without altering genomic sequences. **(E)** CRISPRi: Catalytically inactive dCas9 is fused to the transcriptional repressor domain (e.g., KRAB). Upon binding of the dCas9-repressor complex to the promoter region of the target gene, the KRAB domain recruits repressive chromatin modifiers to suppress target gene transcription, achieving sequence-specific gene silencing. Created with BioRender.com.

**Figure 4:**
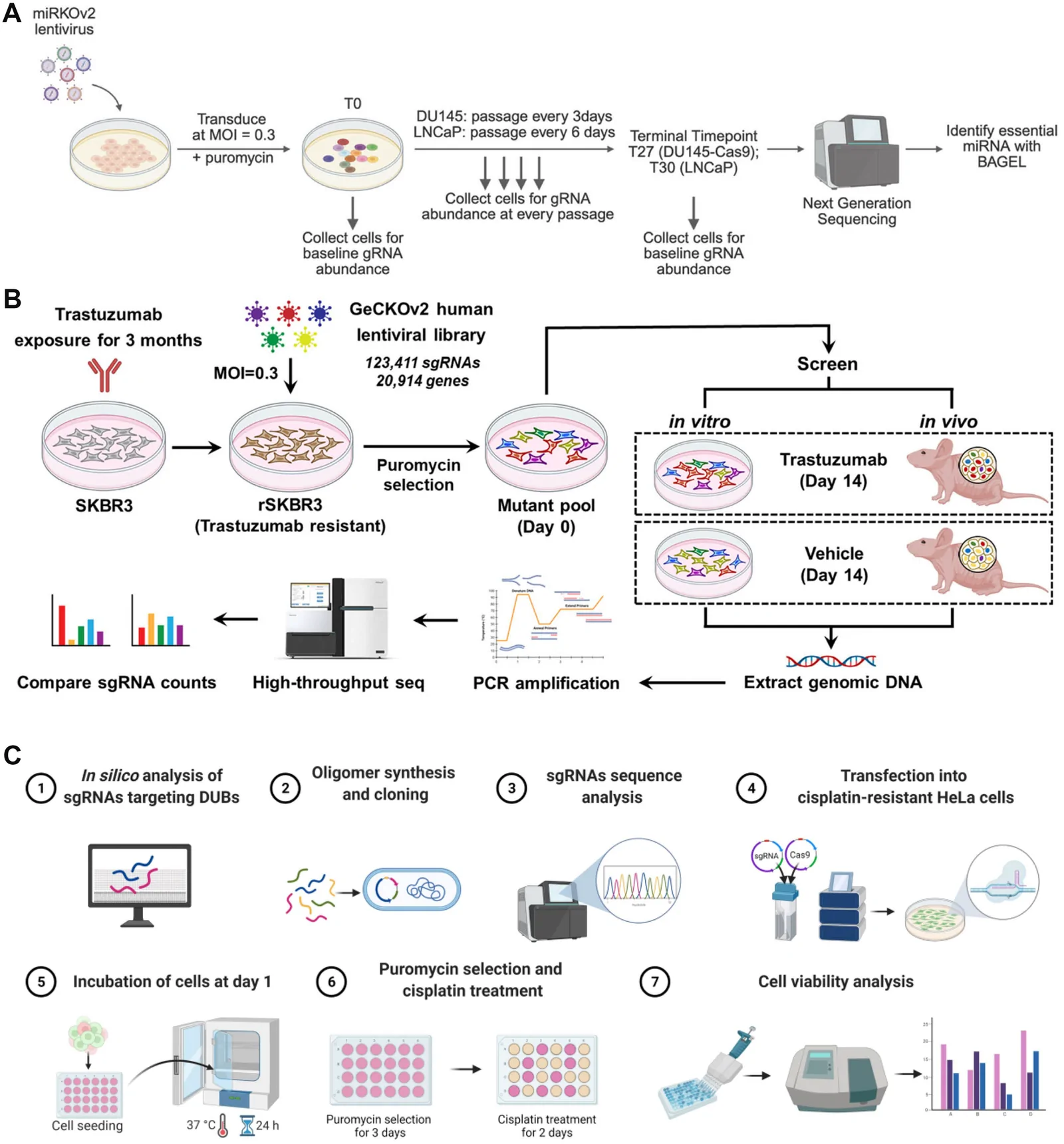
** CRISPR screening and drug resistance research. (A)** Schematic diagram illustrating the identification of essential miRNAs in prostate cancer cells via miRNA-focused CRISPR screening. Adapted with permission from 121, copyright 2025 The Authors. **(B)** Flowchart of a genome-wide CRISPR screening to identify genes linked to anti-HER2 resistance using a pooled human GeCKOv2.0 lentiviral sgRNA library. Adapted with permission from 127, copyright 2022 The Authors. **(C)** Schematic overview of a CRISPR/Cas9-based screening system for cisplatin resistance research. Adapted with permission from 129, copyright 2022 The Authors.

**Figure 5:**
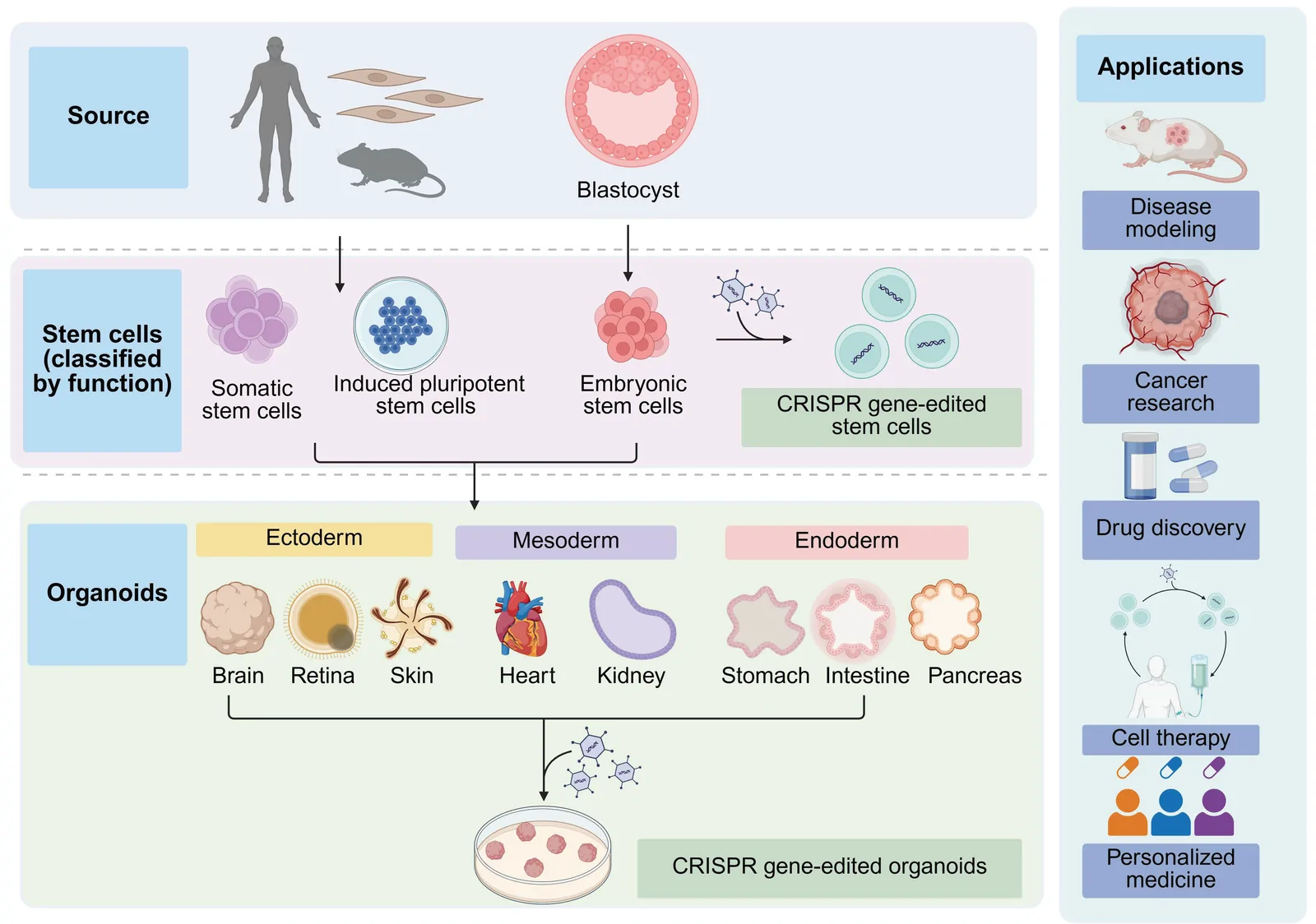
** The applications of human stem cells and organoids.** Stem cell sources include somatic stem cells derived from adult human or rodent tissues, induced pluripotent stem cells (iPSCs) reprogrammed from somatic cells, and embryonic stem cells isolated from the blastocyst. All of these stem cell types can be genetically engineered via CRISPR technology to introduce specific mutations. Pluripotent stem cells differentiate into ectoderm-derived organoids (brain, retina, skin), mesoderm-derived organoids (heart, kidney), and endoderm-derived organoids (stomach, intestine, pancreas), which can also be directly engineered via CRISPR systems. These platforms, particularly CRISPR-modified models, facilitate disease modeling, cancer research, drug discovery, cell therapy, and personalized medicine. Created with BioRender.com.

**Figure 6:**
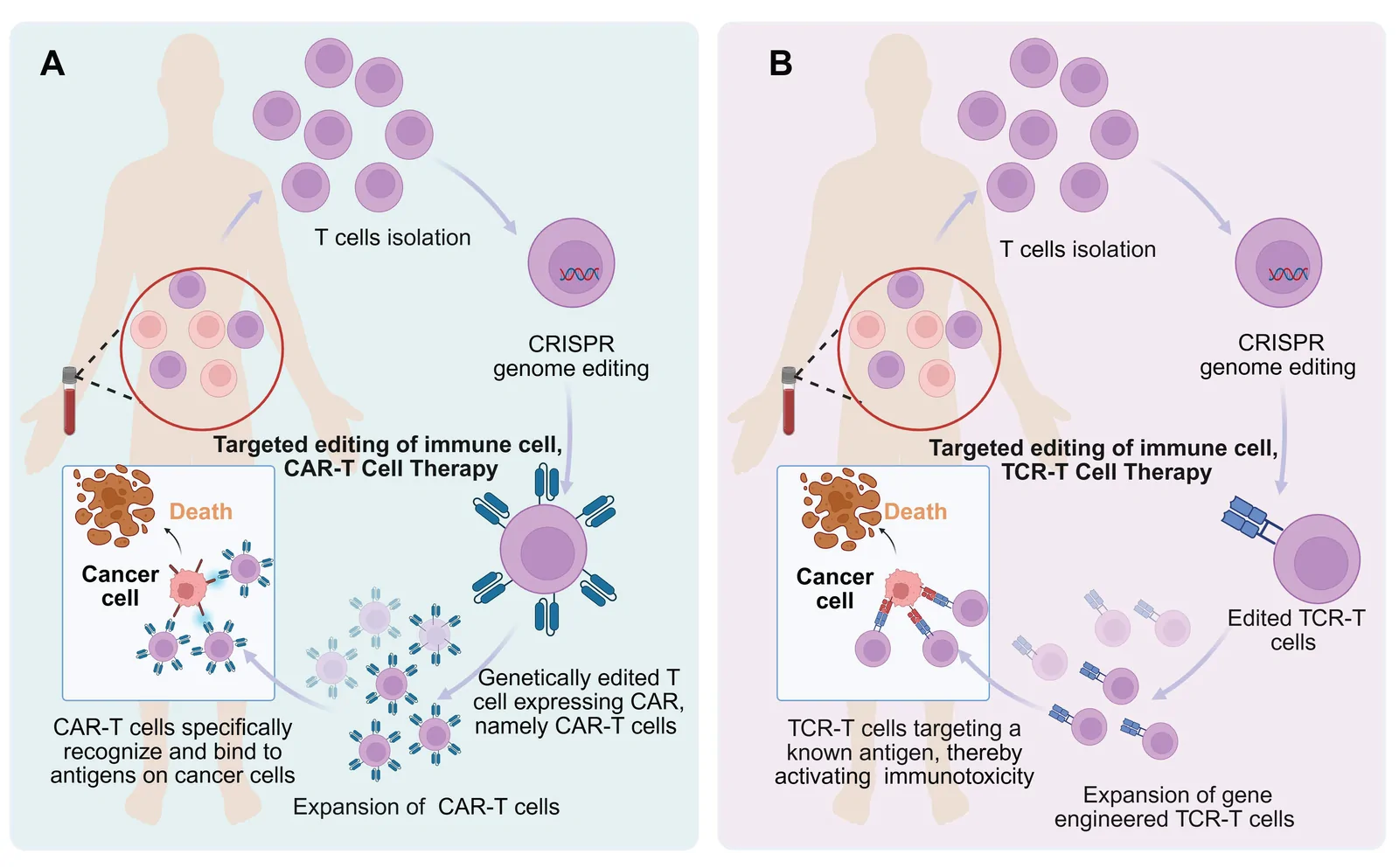
** Genome editing of immune cells using CRISPR. (A)** CAR-T cell therapy; **(B)** TCR-T cell therapy. Created with BioRender.com.

**Figure 7:**
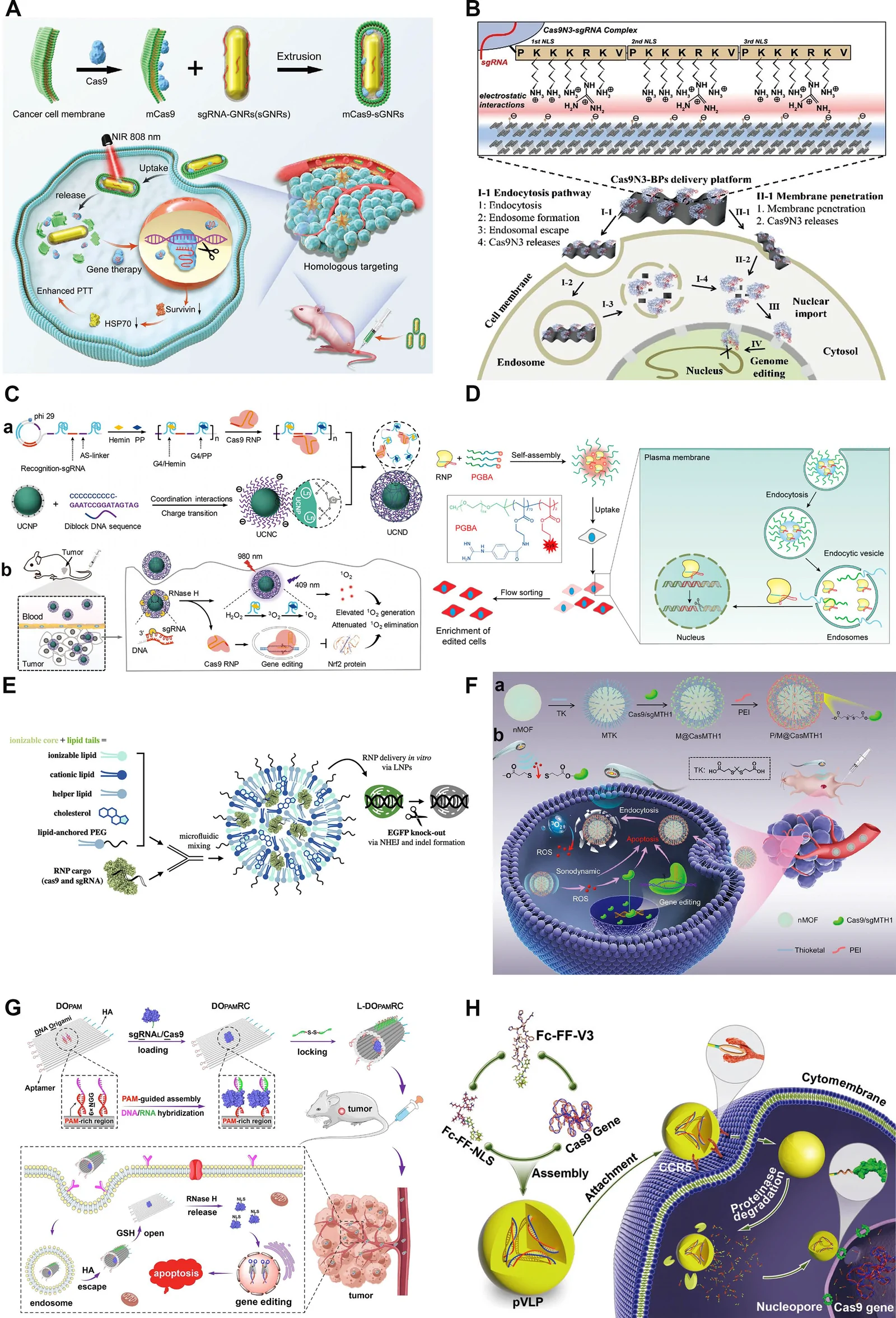
** Representative examples of non-viral delivery approaches for CRISPR. (A)** Synthesis procedures of Cas9-loaded gold nanorods and processes of combined photothermal/gene therapy. Adapted with permission from 282, copyright 2022 John Wiley and Sons. **(B)** Design of black phosphorus nanosheets for delivering CRISPR component and related intracellular delivery pathways. Adapted with permission from 284, copyright 2018 John Wiley and Sons. **(C)** Multifunctional DNA/upconversion nanoparticle complex for synergistic photodynamic therapy by delivering Cas9 RNP, hemin and protoporphyrin. Adapted with permission from 287, copyright 2024 John Wiley and Sons. **(D)** Preparation of PGBA-RNP polymeric NPs and overview of mechanisms for genome editing. Adapted with permission from 294, copyright 2024 American Chemical Society. **(E)** CRISPR/Cas9 RNP-encapsulated LNPs for *in vivo* delivery. Adapted with permission from 235, copyright 2025 American Chemical Society. **(F)** a) Preparation of the nano-sonosensitizer metal–organic frameworks P/M@CasMTH1 and b) ultrasound-triggered gene editing-augmented sonodynamic therapy for oncologic interventions. Adapted with permission from 312, copyright 2021 John Wiley and Sons. **(G)** Design and construction of CRISPR system based on DNA origami for gene therapy. Adapted with permission from 329, copyright 2023 John Wiley and Sons. **(H)** The viral peptide and Cas9 gene were co-assembled into peptidyl virus-like particles capable of transferring genes through the viral entry pathway. Adapted with permission from 237, copyright 2018 John Wiley and Sons.

**Figure 8:**
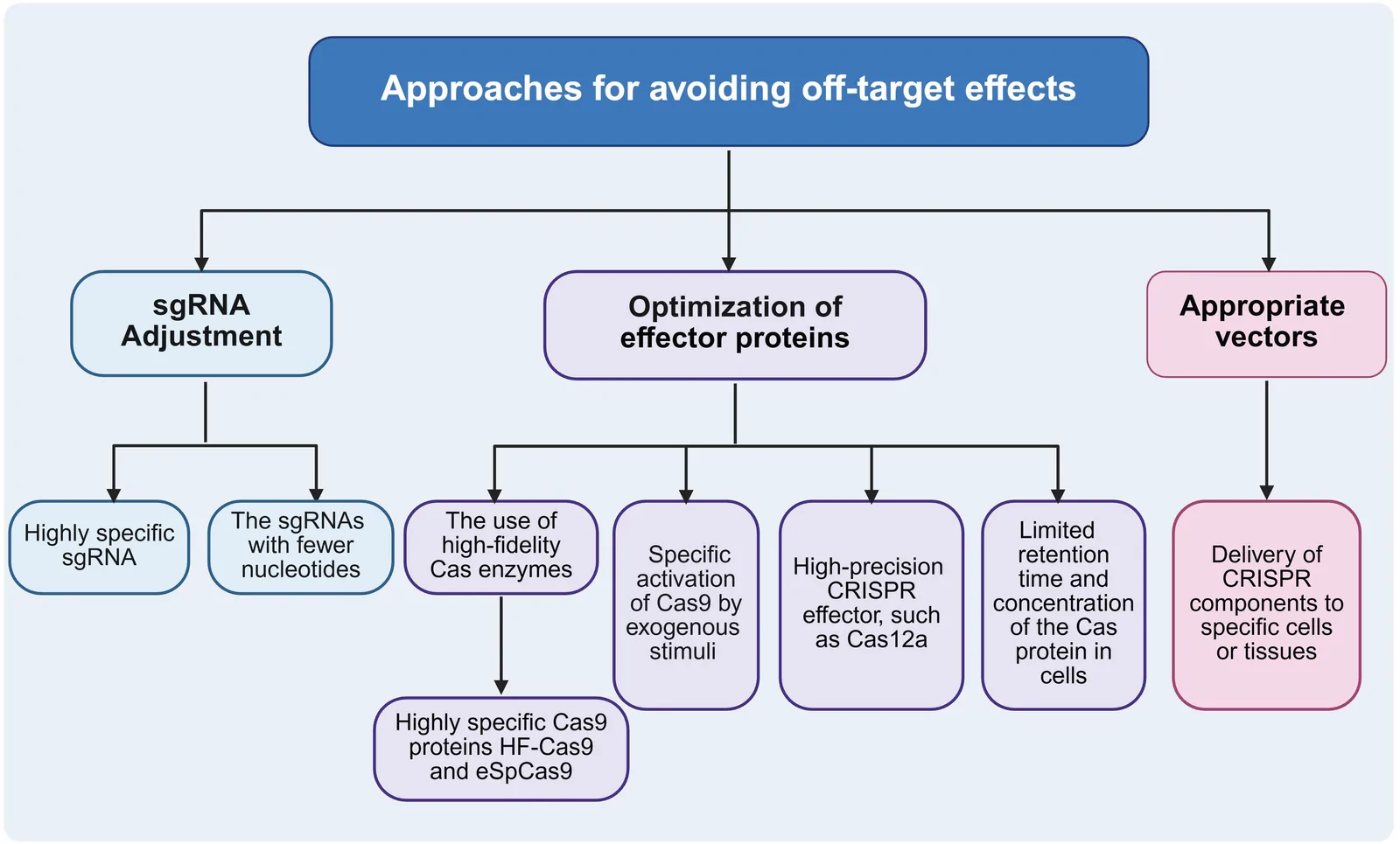
Approaches for avoiding CRISPR off-target effects. Created with BioRender.com.

**Table 1:** Distinctive features of representative Class II Cas effectors in genome editing.

Type	Cas protein	gRNA	PAM requirement	Target type	Collateral cleavage	Characteristics	Refs
Type II	Cas9	crRNA and tracrRNA	Yes, NGG	DNA	No	Most commonly used for gene editing	7,46,51
Type V	Cas12a	crRNA	Yes, TTTV	DNA	ssDNA	High specificity, low off-target effects	40-46
Type VI	Cas13a/b	crRNA	No, PFS	RNA	ssRNA	Post-transcriptional knockdown	46-50

**Table 2:** Main characteristics of base editing and prime editing.

Type	DSB requirement	Editing scope	Main advantages	Limitations	Refs
Base editing	No, single-strand break	Single nucleotide transition	High editing efficiency; low indel rate	Dependent on fixed distance to PAM; bystander edits	12,62,64
Prime editing	No, single-strand break	Small insertions, deletions or base conversions	No exogenous donor DNA template required; low off-target effect	Lower efficiency; reverse transcriptase lacks proofreading activity	13,14,62

**Table 3:** Gene activation and inhibition via CRISPRa and CRISPRi technology.

Type	Function	Domain	Target cell	Delivery vector	Target	Efficiency	Refs
CRISPRa	Reversible gene activation	VP64	MDA-MB-231	Polymeric nanoparticle	miR-524	3-fold *in vivo*	94
		VP64	Astrocytes	Lentivirus, AAV	Ngn2	20-fold* in vitro*	87
		VP64	Astrocytes	Lentivirus, AAV	Isl1	10-fold* in vitro*	87
		VP64	A2780	Polymeric nanoparticle	CT45	4-fold* in vitro*	95
		VP160	C2C12	Plasmid electroporation	Lama1	Not applicable	96
CRISPRi	Reversible inhibition	KRAB	Rod cells	AAV	Nrl	Up to 80% *in vivo*	93
		KRAB	AML-12	AAV	PCSK9	30% *in vivo*	97

**Table 4:** Characteristics of CRISPR/Cas nucleic acid detection.

Category	Traditional detection	CRISPR/Cas detection	Refs
Properties	PCR amplification and detection	Targeted identification and cleavage by CRISPR/Cas system	132,133,139
Equipment requirement	Sophisticated thermocycler and fluorescence detection equipment	Constant temperature condition	134,136
Cost	High	Cost-effective	39
Specificity	High specificity is achieved through primer and probe design	The target sequence is identified by gRNA with high specificity and can be distinguished from single base mutations	135,137,138
Efficiency	Time-consuming and amplification is required	Fast and no complicated thermal cycle steps are required	134,137
Application scenarios	Laboratory testing, high throughput screening	Field testing, resource limited environment	39,137-139

**Table 5:** Overview of gene therapy based on CRISPR technology in clinical trials.

Target locus	ClinicalTrials.gov identifier	Year	Phase	Disease indication	Intervention	Sponsor
*PD-1*	NCT02793856	2016	Phase 1	Lung cancer	Infusion of edited cells	Sichuan University, China
*PD-1*	NCT03747965	2018	Phase 1	Solid tumors	Infusion of edited cells	Chinese PLA General Hospital, China
*PD-1*	NCT04417764	2019	Phase 1	Advanced hepatocellular carcinoma	PD-1 knockout engineered T-cell infusion	Central South University, China
*PD-1*	NCT03081715	2017	Not applicable	Advanced esophageal cancer	Infusion of PD-1 knockout T cells	Hangzhou Cancer Hospital, China
*PD-1*	NCT03044743	2017	Phase 1/2	Advanced stage Epstein‒Barr virus associated malignancies	Infusion of edited cytotoxic T lymphocytes with PD-1 knockout	The Affiliated Nanjing Drum Tower Hospital of Nanjing University Medical School, China
*PD-1 and TCR*	NCT03545815	2018	Phase 1	Solid tumors	CRISPR/Cas9 treated CAR-T cell infusions	Chinese PLA General Hospital, China
*TCR and PD-1*	NCT03399448	2018	Phase 1	Myeloma, sarcoma, melanoma	Infusion of edited cells	University of Pennsylvania, USA
*HPV E6/E7*	NCT03057912	2018	Phase 1	HPV-related cervical intraepithelial neoplasia	Plasmid in gel	First Affiliated Hospital, Sun Yat-Sen University, China
*CLL-1*	NCT06128044	2024	Phase 1	Relapsed/refractory acute myeloid leukemia	Allogeneic CAR-T cell therapy	Caribou Biosciences, Inc., USA
*TGFβR*	NCT04976218	2022	Phase 1	Advanced EGFR positive solid tumors	Infusion of edited cells	Chinese PLA General Hospital, China
*CD5*	NCT04767308	2021	Phase 1	Hematopoietic malignancies	Infusion of edited cells	Huazhong University of Science and Technology, China
*CD33*	NCT04849910	2021	Phase 1/2	Acute myeloid leukemia and myelodysplastic syndromes	Genome edited hematopoietic stem and progenitor cell therapy	Vor Biopharma, USA
*CD33*	NCT05662904	2023	Phase 1	Acute myeloid leukemia	CD33-deleted CD34+ hematopoietic stem cells	German Cancer Research Center, Germany
*CD52, TRAC*	NCT04557436	2020	Phase 1	B-cell acute lymphoblastic leukemia	Infusion of edited allogenic T cells	Great Ormond Street Hospital for Children NHS Foundation Trust, UK
*TRAC, PD1*	NCT04637763	2021	Phase 1	Relapsed/refractory B cell non-Hodgkin lymphoma	CRISPR-edited allogeneic CAR-T cell therapy	Caribou Biosciences, Inc., USA
*TRAC, B2M*	NCT04502446	2020	Phase 1	Relapsed or refractory T-cell or B-cell malignancies	Allogeneic CRISPR/Cas9-engineered T cells	CRISPR Therapeutics AG, Switzerland
*TRAC, B2M*	NCT04244656	2020	Phase 1	Multiple myeloma	Edited allogeneic T cells	CRISPR Therapeutics AG, Switzerland
*TRAC, B2M*	NCT05795595	2023	Phase 1/2	Relapsed or refractory solid tumors	Edited allogeneic T cells	CRISPR Therapeutics AG, Switzerland
*TRAC, B2M*	NCT05722418	2023	Phase 1	Relapsed/refractory multiple myeloma	Allogeneic CAR-T cell therapy	Caribou Biosciences, Inc., USA
*TRAC, B2M*	NCT05643742	2023	Phase 1/2	Relapsed or refractory B-cell malignancies	Edited allogeneic T cells	CRISPR Therapeutics AG, Switzerland
*CISH*	NCT04426669	2020	Phase 1/2	Metastatic gastrointestinal cancers	Autologous *CISH* inactivated tumor infiltrating lymphocytes	Intima Bioscience, Inc., USA
*CISH*	NCT05566223	2023	Phase 1/2	Metastatic non-small cell lung cancer	Autologous *CISH* inactivated tumor infiltrating lymphocytes	Intima Bioscience, Inc., USA
*BCL11A*	NCT03655678	2018	Phase 2/3	TDT	Infusion of edited cells (CTX001)	Vertex Pharmaceuticals Incorporated, USA
*BCL11A*	NCT03745287	2018	Phase 2/3	Severe SCD	Infusion of edited cells (CTX001)	Vertex Pharmaceuticals Incorporated, USA
*BCL11A*	NCT05356195	2022	Phase 3	Pediatric participants with TDT	Infusion of edited cells (CTX001)	Vertex Pharmaceuticals Incorporated, USA
*BCL11A*	NCT05329649	2022	Phase 3	Pediatric participants with severe SCD	Infusion of edited cells (CTX001)	Vertex Pharmaceuticals Incorporated, USA
*BCL11A*	NCT05477563	2022	Phase 3	TDT or severe SCD	Infusion of edited cells (CTX001)	Vertex Pharmaceuticals Incorporated, USA
*BCL11A*	NCT06300723	2024	Not applicable	SCD	CD34+ autologous hematopoietic stem and progenitor cells edited at the *BCL11A* gene	Bioray Laboratories, USA
*BCL11A*	NCT05951205	2024	Phase 3	Adolescent and adult participants with severe SCD, βS/βC genotype	Infusion of edited cells (CTX001)	Vertex Pharmaceuticals Incorporated, USA
*HBB*	NCT03728322	2018	Phase 1	Thalassemia	Infusion of edited cells	Allife Medical Science and Technology Co., Ltd., China
*HBG1/2*	NCT06041620	2023	Not applicable	TDT	Autologous CRISPR/Cas12b edited hematopoietic stem cells	Institute of Hematology & Blood Diseases Hospital, China
*F9*	NCT06379789	2024	Phase 1/2	Hemophilia B	CRISPR/Cas9 based *F9* gene insertion therapy administered via intravenous infusion	Regeneron Pharmaceuticals, USA
*OTOF*	NCT06025032	2024	Phase 1	Hearing loss	CRISPR/Cas13 RNA base editing therapy administered via intracochlear injection	HuidaGene Therapeutics Co., Ltd., China
*DMD*	NCT05514249	2022	Phase 1	DMD	CRD-TMH-001 administered via intravenous injection	Cure Rare Disease, Inc, USA
*PCSK9*	NCT05398029	2022	Phase 1	Heterozygous familial hypercholesterolemia, atherosclerotic cardiovascular disease, and uncontrolled hypercholesterolemia	Disrupted expression of the *PCSK9* gene by base editing technology via intravenous infusion	Verve Therapeutics, Inc., USA
*CEP290*	NCT03872479	2019	Phase 1/2	Leber congenital amaurosis 10	EDIT-101 administered via subretinal injection	Editas Medicine, Inc., USA
*VEGFA*	NCT06031727	2023	Phase 1	Neovascular age-related macular degeneration	Cas13 technology using one single AAV vector upon subretinal injection	HuidaGene Therapeutics Co., Ltd., China
*MYOC*	NCT06465537	2024	Not applicable	Primary open-angle glaucoma	Intracameral injection of virus-like particle	Shanghai BDgene Co., Ltd., China
*CCR5*	NCT03164135	2017	Not applicable	HIV-1	Infusion of edited cells	Affiliated Hospital of the Academy of Military Medical Sciences, China
*HIV-1*	NCT05144386	2022	Phase 1	Aviremic HIV-1 infected adults	CRISPR/Cas9 gene editing system delivered by AAV9	Excision BioTherapeutics, USA

**Table 6:** Common characteristics of CRISPR viral delivery methods.

Delivery vector	Type	Most common cargo	Advantages	Limitations	Refs
AAV	Non-enveloped	~4.7 kb, ssDNA	Efficient delivery; different serotypes can target specific tissues	Low packaging capacity; preexisting immunity	117,204,251,252
AdV	Non-enveloped	8 kb-36 kb, dsDNA	No genomic integration; good genetic capacity	Inflammatory response; nonpersistent gene-editing effects	252-260
LV	Enveloped	~10 kb, ssRNA	Persistent genetic effects; low immunogenicity	Genomic integration; low efficiency *in vivo*	161,252,261-267
